# Evolutionary analyses of the gasdermin family suggest conserved roles in infection response despite loss of pore-forming functionality

**DOI:** 10.1186/s12915-021-01220-z

**Published:** 2022-01-07

**Authors:** Diego Angosto-Bazarra, Cristina Alarcón-Vila, Laura Hurtado-Navarro, María C. Baños, Jack Rivers-Auty, Pablo Pelegrín

**Affiliations:** 1 Línea de Inflamación Molecular, Instituto Murciano de Investigación Biosanitaria IMIB-Arrixaca, Hospital Clínico Universitario Virgen de la Arrixaca, Carretera Buenavista s/n. 30120 El Palmar, Murcia, Spain; 2 Tasmanian School of Medicine, University of Tasmania, Tasmania, Australia; 3 Department of Biochemistry and Molecular Biology B and Immunology, Faculty of Medicine, University of Murcia, 30120 Murcia, Spain

**Keywords:** Pyroptosis, Gasdermin, Pejvakin, Evolution, Sepsis, Infection

## Abstract

**Background:**

Gasdermins are ancient (>500million-years-ago) proteins, constituting a family of pore-forming proteins that allow the release of intracellular content including proinflammatory cytokines. Despite their importance in the immune response, and although gasdermin and gasdermin-like genes have been identified across a wide range of animal and non-animal species, there is limited information about the evolutionary history of the gasdermin family, and their functional roles after infection.

In this study, we assess the lytic functions of different gasdermins across Metazoa species, and use a mouse model of sepsis to evaluate the expression of the different gasdermins during infection.

**Results:**

We show that the majority of gasdermin family members from distantly related animal clades are pore-forming, in line with the function of the ancestral proto-gasdermin and gasdermin-like proteins of Bacteria. We demonstrate the first expansion of this family occurred through a duplication of the ancestral gasdermin gene which formed gasdermin E and pejvakin prior to the divergence of cartilaginous fish and bony fish ~475 mya. We show that pejvakin from cartilaginous fish and mammals lost the pore-forming functionality and thus its role in cell lysis. We describe that the pore-forming gasdermin A formed ~320 mya as a duplication of gasdermin E prior to the divergence of the Sauropsida clade (the ancestral lineage of reptiles, turtles, and birds) and the Synapsid clade (the ancestral lineage of mammals). We then demonstrate that the gasdermin A gene duplicated to form the rest of the gasdermin family including gasdermins B, C, and D: pore-forming proteins that present a high variation of the exons in the linker sequence, which in turn allows for diverse activation pathways. Finally, we describe expression of murine gasdermin family members in different tissues in a mouse sepsis model, indicating function during infection response.

**Conclusions:**

In this study we explored the evolutionary history of the gasdermin proteins in animals and demonstrated that the pore-formation functionality has been conserved from the ancient proto-gasdermin protein. We also showed that one gasdermin family member, pejvakin, lost its pore-forming functionality, but that all gasdermin family members, including pejvakin, likely retained a role in inflammation and the physiological response to infection.

**Supplementary Information:**

The online version contains supplementary material available at 10.1186/s12915-021-01220-z.

## Background

The gasdermin family of pore-forming proteins was initially identified in the gastrointestinal tract and dermis of mammalian species [1, 2]. However, gasdermin family members have now been identified in a wide range of cell types and tissues in a vast array of organisms across the animal kingdom [3, 4]. Gasdermins regulate a type of inflammatory programmed cell death called pyroptosis, characterized by the permeabilization of the plasma membrane [3, 4]. Six gasdermin genes have been identified in the human genome, with five named gasdermin A to E and a sixth named pejvakin (PJVK).

Gasdermin proteins share a highly conserved N-terminal domain, that upon release from the repressor C-terminus, are able to bind to lipids of the plasma membrane and form homo-oligomeric pores, disrupting cellular ionic balance and inducing osmotic cell swelling [5, 6]. The N-termini of gasdermin D (GSDMD) and gasdermin E (GSDME) have also been found to target organelle membranes such as the mitochondria [7].

To date, the activation mechanism described for the different gasdermins relies on the proteolytic cleavage of the linker sequence between the N- and C-terminal domains, which releases the N-terminal domain and subsequently induces pyroptosis [5, 6, 8]. In mammalian species, the cleavage, and thus activation, of the gasdermin family members occurs through a range of proteases, including caspase-3 cleaving GSDME, caspase-1 and granzyme A cleaving gasdermin B (GSDMB), and caspase-1/4/5/8/11, neutrophil proteases and cathepsin cleaving GSDMD [5, 6, 9–14]. The upstream signaling that controls the activation of these proteases is diverse, and increasing our understanding of these processes is important from a human health perspective as the activation of these pathways has been linked to a range of inflammatory pathological processes [5, 6, 9, 15]. A recent focus of research in this area is the activation of caspase-1 by inflammasomes, which are cytosolic multiprotein oligomers formed by the activation of a range of intracellular pattern recognition receptors through canonical pathways or non-canonical pathways facilitated by caspases 4/5 (human) or 11 (murine).

Inflammasome-induced GSDMD N-terminal membrane pore formation is followed by the release of the bioactive forms of the proinflammatory cytokines interleukin (IL)-1β and IL-18 [16–18]. Gasdermin D-mediated pyroptosis also induces the release of different intracellular molecules implicated in the inflammatory response, known as damage-associated molecular patterns, and includes the high-mobility group box 1 or inflammasome oligomers [19–21]. Additionally, GSDMD activation in neutrophils contributes to the extrusion of nuclear DNA during the formation of bactericidal neutrophil-derived extracellular traps (NETs); this is a unique form of cell death called NETosis [10, 22].

In humans, single-nucleotide polymorphisms in hs-*gsdma* and hs-*gsdmb* have been associated with asthma [23], while mutations in hs-*gsdme* and hs-*pjvk* are associated with non-syndromic deafness [24, 25]. In mice, the pejvakin protein (mm-PJVK) is able to affect the autophagy of peroxisomes in auditory hair cells and protect them against damage, which explains its role in deafness [26, 27]. However, while ectopic expression of the N-terminus of hs-GSDMA to E can permeabilize the plasma membrane [5], it is not well established if human PJVK or PJVK from other species can also do the same [28], as it is considered an ancient member of the gasdermin family [4, 29].

In this study, we show that pejvakin and gasdermin E represent the first expansion of the gasdermin gene family between 450 and 500 million years ago prior to the divergence of the jawed-vertebrate clade. We map the evolution of pore formation in gasdermin family members using cellular expression systems and demonstrate that pejvakin cannot generate pores from disparately related species. We also propose that the reduced purifying selection pressure resulted in accumulated mutations in pejvakin to the point where it lost its ancestral function of pore formation. Studies of the structural and functional homology of mouse, lancelet, shark, and bony fish gasdermins reveal that non-Chordata gasdermins have limited lytic functionality, but that they are able to induce IL-1β release. GSDMA formed from a duplication event of GSDME in the common ancestor of the Amniotes (Aves, Reptilia, and Mammalia). This was followed by an expansion of the gasdermin family in the mammalian common ancestor to form the gasdermin A subfamily, which in turn allowed for diverse pathways to control and execute pyroptosis due the high variability of exons coding for the linker sequence between the N- and C-terminal domains. Finally, we demonstrate that there was significant modulation of the expression profiles of all murine gasdermins in a wide range of tissues during infection, suggesting there could be some functional conservation in this family in regulating the host infection response.

## Results

### Gasdermin protein family evolution: from the Precambrian to the mammalian expansion

**Fig. 1:**
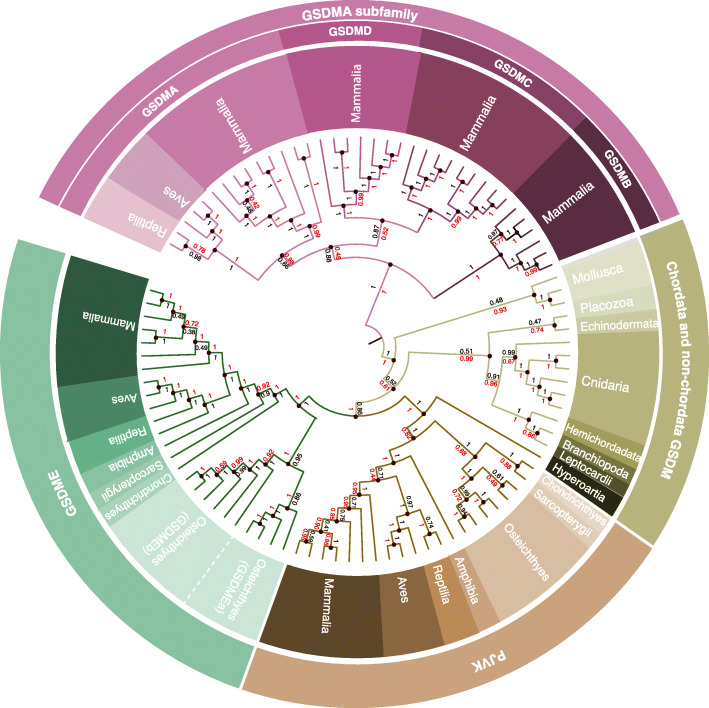
Phylogeny of the gasdermin family. Phylogenetic tree inferred from amino acid sequence data using the Bayesian (black numbers) and maximum likelihood (red numbers) methods showing divergence of full-length gasdermins in mammals, birds, reptiles, amphibians, fish, jawless Chordata, and non-Chordata. Black numbers show the posterior distribution of the Bayesian method and red numbers show the bootstrap analysis of the maximum likelihood analysis

Using specific gasdermin full-length amino acid sequences as queries in the different available databases, including both BLASTP and BLASTN searches, we found one or more gasdermin sequences in a wide variety of species (Additional file 1: Table S1). The ubiquitous nature of gasdermins across the breadth of the Metazoa suggests that the founding member of the gasdermin family arose as a single proto-gasdermin gene (Fig. 1) in the common ancestor of all Metazoa prior to the Cambrian explosion ~540 million-years-ago (mya). Interestingly, we could not identify gasdermin sequences in the Ecdysozoa clade (including Nematoda or Arthropoda) genomes. Given that the divergence of Ecdysozoa occurred after the evolution of the proto-gasdermin gene in a common ancestor of the Metazoa, we propose that the common ancestor of the Ecdysozoa clade lost the proto-gasdermin gene [30] (Additional file 1: Fig. S1A). In agreement with De Schutter et al. [29], the phylogeny from the Bayesian tree shows how the gasdermin sequences from invertebrates are separate from the PJVK and GSDME sequences from vertebrates, these two sharing the same node (Fig. 1). However, in the maximum likelihood analysis, we found gasdermins from invertebrates sharing the same node with the PJVK sequence from vertebrates (Additional file 1: Fig. S2). N-terminal gasdermin-like genes found in bacteria and fungi, named *regulated cell death-1* (*rcd-1*) in fungi, have been reported to induce cell lysis by membrane binding [31, 32]; however, *rcd-1* genes share no relevant sequence identity with invertebrate and vertebrate gasdermin sequences, suggesting these gene families are evolutionarily distinct analogues (Additional file 1: Fig. S1B).

As all jawed vertebrates (Gnathostomata) have both GSDME and PJVK sequences and neither bony nor cartilaginous fish (*Osteichthyes* and *Chondrichthyes*, respectively) have any other homologs to the ancestral gasdermin, we conclude that the first expansion of the gasdermin family occurred through a gene duplication event of the ancestral gasdermin gene which formed GSDME and PJVK prior to the divergence of *Chondrichthyes* and *Osteichthyes* between 450 and 500 mya [33] (Fig. 1). In Teleostei bony fishes, there were two *gsdme* genes, named *gsdmea* and *gsdmeb* (Fig. 1). The lack of these two *gsdme* genes in non-Teleostei bony fishes such as Holostei, and the conservation of synteny of *gsdmea* and *gsdmeb* (discussed below) suggest that these were a result of whole genome duplication of Teleostei bony fishes that occurred approximately 320 mya [34].

**Fig. 2:**
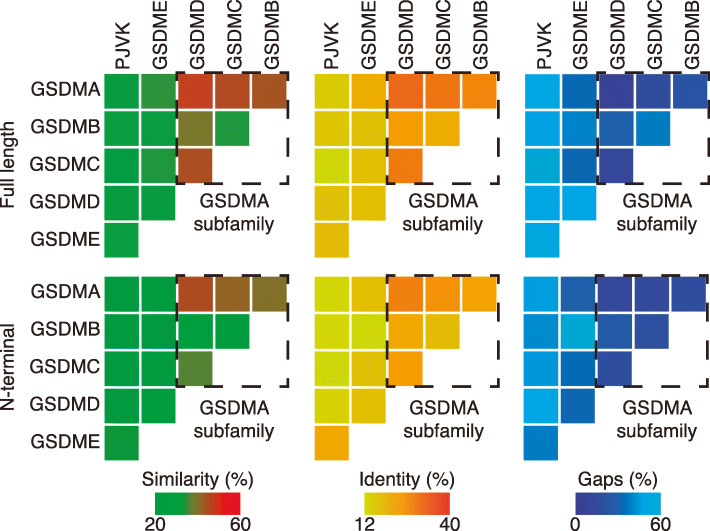
Human gasdermin sequence similarity. Heat maps showing the percentage of similarity (left), identity (middle), and gaps (right) between the full-length gasdermin sequences (top) and their respective N-terminal domain (bottom). GSDMA subfamily is highlighted with a dashed line box and presents the higher similarity

Previous studies have proposed the exclusion of PJVK from the gasdermin family partly on the basis of the comparatively heavily truncated C-terminal domain of the protein [2]. However, our phylogenetic analyses strongly suggest that PJVK evolved from a common ancestral gasdermin gene (Fig. 1 and 2, Additional file 1: Fig. S2) Furthermore, the sequence identity between *Homo sapiens* PJVK and GSDME (32.8%) is higher than that between GSDME and other family members such as GSDMA (26.4%), indicating that PJVK cannot reasonably be excluded from the gasdermin family (Fig. 2) and that PJVK could have only emerged by the duplication of an ancestral *gsdme* gene [29].

GSDMA was found in birds (Aves), reptiles (Reptilia), and mammals (Mammalia), and this, along with sequence identity evidence, suggests that GSDMA formed from a gene duplication event of GSDME in the common ancestor of the Amniotes after the divergence of the Lissamphibians and prior to the divergence between the Sauropsids (ancestral lineage of reptiles, turtles, and birds) and Synapsids (ancestral lineage of mammals) ~320 mya. A higher sequence identity was present among gasdermins A, B, C, and D (Fig. 2), and these gasdermins are only found in mammals. This suggests GSDMA underwent a number of duplication events in the Synapsid mammalian common ancestor which gave rise to gasdermins B, C, and D between ~320 and ~160mya (Fig. 1). However, while unlikely, it is possible that one or more of these gasdermins (B, C, and D) were present in the Amniote common ancestor and were lost in the Sauropsida lineage prior to the divergence of the Aves and Reptilia lineages (Fig. 1 and Additional file 1: Fig. S2).

### Evolutionary relationship between gasdermin protein family members

**Fig. 3:**
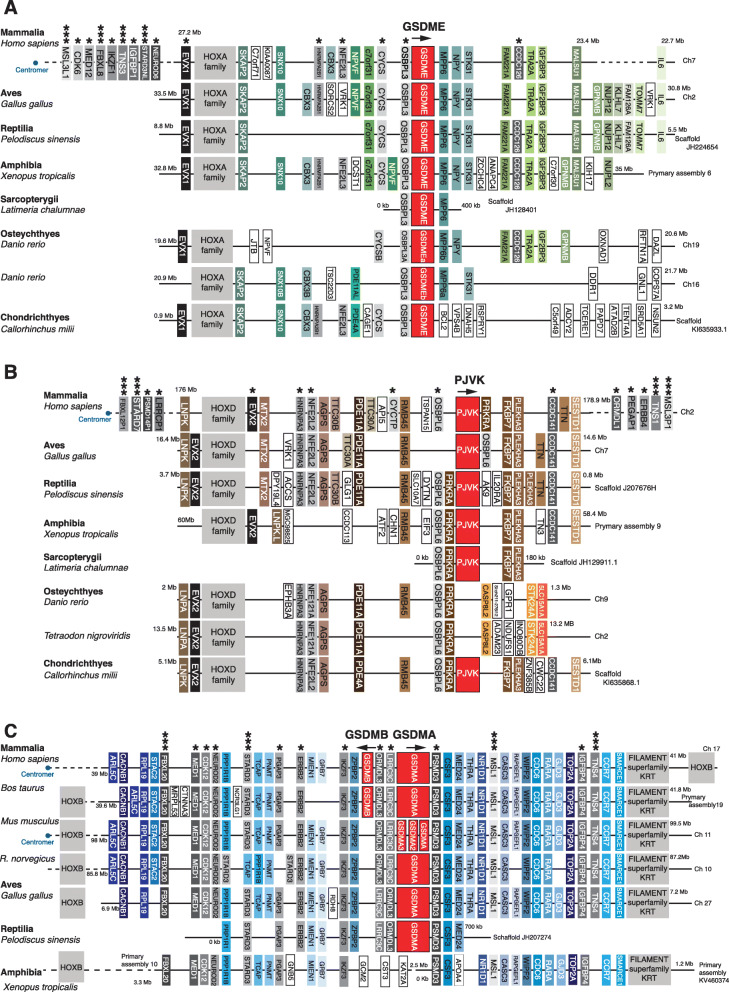
Synteny analysis of the gasdermin E, pejvakin, and gasdermin A/B loci from different species. Schematic diagrams showing the conservation of synteny in the *gsdme* loci **A**, *pjvk* loci **B**, and *gsdma*/*b* loci **C** in Mammalia (*Homo sapiens*, *Mus musculus*, *Rattus norvegicus, Bos taurus*), Aves (*Gallus gallus*), Reptilia (*Pediscus sinensis*), Amphibia (*Xenopus tropicalis*), and fish (Osteychthyes: *Latimeria chalumnae, Danio rerio, Tetraodon nigroviridis*, and Chondrichthyes: *Callorhinchus milii*). In **B**, for *Danio rerio,* the synteny of the genes surrounding the mammalian *pjvk* is presented to illustrate that fish *pjvk* is not located in that part of the genome. In **C**, amphibian synteny of genes surrounding mammalian *gsdma* gene is shown to illustrate that no *gsdma* is present. Conserved genes are indicated in different colors and the direction of gasdermin gene transcription is indicated with arrows. The position of the centromeres is indicated with a blue dot. An asterisk denotes genes from the same family found in two of the genomic environments (*gsdme*/*pjvk*, *gsdme*/*gsdma,* or *pjvk*/*gsdma*); three asterisks denote genes from the same family found in the three genomic environments

**Fig. 4:**
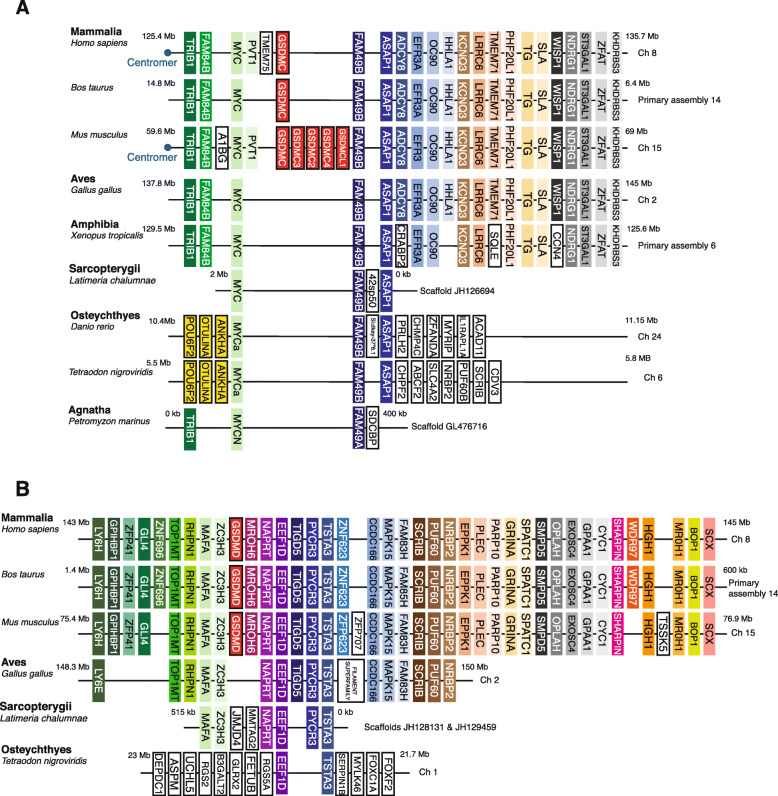
Synteny analysis of the gasdermin C and D loci from different species. Schematic diagrams showing the conserved synteny in the *gsdmc* loci (**A**) and *gsdmd* loci (**B**) in Mammalia (*Homo sapiens*, *Mus musculus, Bos taurus*). The synteny of the genes surrounding the mammalian *gsdmc/d* is shown in Aves (*Gallus gallus*), Amphibian (*Xenopus tropicalis*), Fish (*Latimeria chalumnae, Danio rerio, Tetraodon nigroviridis*), and Agnatha (*Petromyzon marinus*) to illustrate that no *gsdmc* or *gsdmd* is present in these species. Conserved genes are indicated in different colors, and the direction of gasdermin gene transcription is indicated with arrows. The position of the centromeres is indicated with a blue dot

In humans, five gasdermins and one *pjvk* gene reside in chromosomes 2q31.2 (hs-*pjvk*), 7p15.3 (hs-*gsdme*), 8q24 (8q24.21 for hs-*gsdmc*, 8q24.3 for hs-*gsdmd*), and 17q21.1 (hs-*gsdma*, hs-*gsdmb*) (Figs. 3 and 4). Gasdermin E and hs-*pjvk* were found adjacent to conserved genes in their proximal genomic environment, including the presence of *hox* cluster of genes (hs-*hoxa* adjacent to hs-*gsdme* and hs-*hoxd* adjacent to hs-*pjvk*) and genes such as hs-*nfe2l2*, hs-*nfe2l3*, hs-*osbpl6*, and hs-*osbpl3* (Fig. 3A, B). Meanwhile, hs-*gsdme* appeared between the hs-*osbpl3* and hs-*ccdc126* genes and hs-*pjvk* was between the hs-*osbpl6* and hs-*ccdc141* genes (Fig. 3A, B), further supporting the evidence that hs-*pjvk* and hs-*gsdme* evolved from a common ancestor during a duplication event of a multigene segment of the chromosome. Furthermore, it is likely that these proteins still retain a functional similarity as is evidenced by the fact that gain-of-function mutations in *gsdme and pjvk* genes have both been associated with non-syndromic deafness. This is the reason for these genes having the alternative names of *dfna5* (*gsdme*) and *dfnb59* (*pjvk*).

In the genetically sequenced non-Chordata species that we analyzed, we found no conservation of synteny proximal to the gasdermin gene loci (Additional file 1: Fig. S3). Only the gasdermin adjacent *nub1* gene is conserved in the genome of *Lingula anatina* (Brachiopoda) and *Lottia gigantea* (Mollusca) (Additional file 1: Fig. S3). However, the other Mollusca species analyzed (*Octopus bimaculoides*) did not conserved the *nub1* gene nor other genes near the *gsdm* gene (Additional file 1: Fig. S3). The lack of synteny conservation proximal to the *gsdm* gene loci makes it difficult to make strong inferences about the evolutionary relationships of gasdermin genes between Chordata and non-Chordata, and within non-Chordata.

Interestingly, the genomic synteny around the *pjvk* gene was conserved in jawed vertebrates in contrast to the other vertebrates, except for bony fishes (Osteichthyes) where no *pjvk* gene was found in that region of the genome (Fig. 3B). Instead, in bony fishes *pjvk* appeared in a different genomic environment surrounded by the *pwp2h* and *col5a2b* genes (Additional file 1: Fig. S4A), indicating that in the bony fishes, lineage *pjvk* translocated to a distinct genome environment.

The *gsdma* genomic synteny was found to be highly conserved in Amniotes, with conserved proximal genes including *lrrc3c* and *psmd3* (Fig. 3C). In Amphibia, where no orthologues of *gsdma* were found, the proximal genes of *lrrc3c*, *ormdl3*, and *psmd3* were highly conserved when compared to Reptilia, Aves, and Mammalia, but no *gsdma* sequence was found in that loci (Fig. 3C), indicating the emergence of the *gsdma* gene in the common ancestor of Reptilia, Aves, and Mammalia (Amniote). Interestingly, *gsdma* was in the same chromosome as the *hoxb* cluster of genes and close to different genes from the same family found in the chromosomes carrying *gsdme* and *pjvk* (Fig. 3A–C). However, compared to the other species analyzed, in humans the hs-*hoxb* was differentially positioned in relation to hs-*gsdma* when compared to *hoxa* and *hoxd* and their positions in relation to *gsdme* and *pjvk*, respectively (Fig. 3A–C). *Mus musculus* presented a triplication of the *gsdma* gene (mm-*gsdma*) in the same genomic environment and were surrounded by the mm-*lrrc3c* and mm-*psmd3* genes (Fig. 3C); however, this was not found in the *Rattus norvegicus* species indicating that this happened recently in evolution after the divergence between mice and rats.

Mammalian organisms presented three specific gasdermin genes, *gsdmb*, *gsdmc*, and *gsdmd* that are not found in other classes of Vertebrata (Figs. 3C and 4A, B). The synteny of proximal genes to *gsdmb* indicates that it appeared as a clear duplication of the *gsdma* gene in the mammalian common ancestor (of the Synapsid clade) (Fig. 3C). The mammalian *gsdmc* and *gsdmd* genes had highly conserved genomic synteny in the species analyzed; however, the proximal genes were different when compared to the other gasdermins and no link to *hoxc* genes was seen (Fig. 4A, B). Phylogenetic tree and sequence identity analyses strongly indicate that *gsdmd* evolved from duplications of the *gsdma* gene (Figs. 1 and 2), placing the *gsdmd* gene in the *gsdma* subfamily. *M. musculus* presented five copies of the mm-*gsdmc* gene (Fig. 4A), but this duplication was not present in the *R. norvegicus* species, again indicating this is a recent event in *M. musculus* evolution. To confirm that *gsdmc* and *gsdmd* genes were not present in non-Mammalia species, we performed whole genome sequence searches and made more detailed interrogations of the sequences of the regions of the genome with high synteny conservation of *gsdmc* and *gsdmd* gene. These searches did not reveal any gasdermin-like sequences (Fig. 4A, B). This analysis indicates that all the gasdermin genes shared the same common ancestral gene which underwent numerous chromosome and gene duplications and local segmental rearrangements to generate the breadth of gasdermin genes we see today (Additional file 1: Fig. S4B).

### Sequence and structural homology of the gasdermin family members

**Fig. 5:**
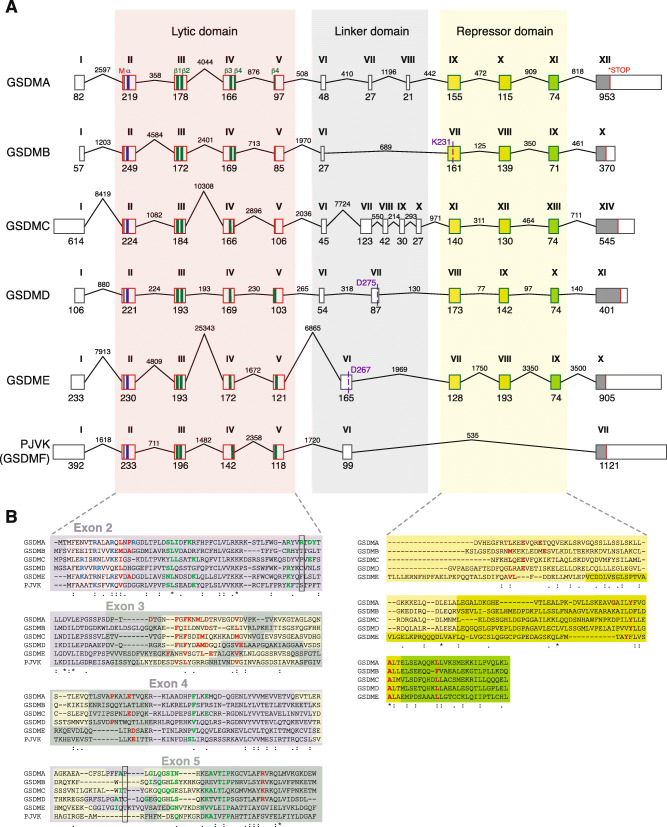
Analysis of the exons and introns present in human gasdermin genes. **A** Schematic representation of the different exons and introns present in the genomic DNA of the different human gasdermins. The first methionine is indicated with a black line, the stop codon with a red line, the first α-helix of the N-terminal domain is indicated with a blue bar, and the four β-sheets that integrate into membranes are indicated with green bars. The exons of the repressor C-terminal domain are represented with different colors to show the conserved similarity between them. Cleavage sites for granzyme A (K^231^) in human GSDMB, caspase-1 (D^275^) in human GSDMD and caspase-3 (D^267^) in human GSDME are shown. For human *gsdmb*, the *gsdmb-403* splice variant is shown. **B** Protein alignment of the N-terminal and repressor C-terminal domains with the different exons highlighted. The residues that are part of the α-helix are highlighted in yellow, and the positive residues involved in the lipid binding are presented in blue. The residues involved in the insertion into membranes are presented in green and the residues involved in the oligomerization of different subunits to form the pore are presented in red. The positions of the Cys^51^ and Cys^191^ of human GSDMD are shown by a black box. In the C-terminal, the residues presented in red are important for the auto-inhibition

Evolution of the gasdermin family was also confirmed by studying the exon/intron composition of the different gasdermin genes. The N-terminal fragment of all gasdermin genes was composed of four different exons that were conserved in all gasdermins, including hs-*pjvk* (Fig. 5). These exons conserved the main secondary structure features of the N-terminal lytic structure, these being the initial α-helix in exon II, the β-sheets 1 and 2 in the exon III, and β-sheets 3 and 4 in exons IV and V (Fig. 5A). In hs-*gsdma*, hs-*gsdmb*, and hs-*gsdmc*, the fourth β-sheet was split between exons IV and V (Fig. 5A). Three conserved exons encoded for the C-terminal repressor domain (Fig. 5A). However, hs-*pjvk* lacked these three exons and therefore its C-terminus was considerably shorter than that of the other gasdermins (Fig. 5A). The loss of these exons was seen in *pjvk* genes from distantly related species (Additional file 1: Fig. S5A), suggesting that the gene duplication event which resulted in *gsdme* and *pjvk*, may have been an incomplete duplication which resulted in the loss of the repressor domain in *pjvk*. Alternatively, the duplication resulted in reduced purifying selection pressure which allowed the accumulation of mutations that included the exon deletions observed (Fig. 5A).

Between the N- and C-terminal domains of the gasdermin, the central linker region presented highly variable compositions in exons and introns, ranging from one single exon (as in hs-*gsdmb*, hs-*gsdme*, or hs-*pjvk*), to two, three, or five exons for hs-*gsdmd*, hs-*gsdma*, and hs-*gsdmc,* respectively (Fig. 5A). This central linker region includes caspase processing sites for hs-GSDMD and hs-GSDME proteins (Fig. 5A). The variation of exons in this central linker region of the gene is responsible for the introduction of different processing sites for proteases to control the activation of the distinct gasdermins. The granzyme cleavage site in the hs-GSDMB protein was at the beginning of the exon VII in the repressor C-terminus. Cleavage results in the release and conformational activation of the lytic hs-GSDMB^NT^ domain (Fig. 5A). Different splice variants of the human *gsdmb* gene have been reported, but all of them modified the presence/absence of exons in the intermediate linker region, thus maintaining the N- and C-terminal exon composition (Additional file 1: Fig. S5B), except for the variant hs-*gsdmb-394* that also affected exons I and II without affecting the N-terminal lytic secondary structure (Additional file 1: Fig. S5B). All four human *gsdmb* splice variants conserved a granzyme sensitive Lysine (K^231^, K^222^, K^244^, amd K^235^ in the first exon of the C-terminal repressor domain), which indicates the functional importance of the cleavage site. One splice variant included an extra exon incorporating a caspase-1 processing site (EEKD^236^ for *GSDMB-416*) [35] (Additional file 1: Fig. S5B), meaning that caspase-1 processing of hs-GSDMB protein was restricted to cells expressing this isoform. In distantly related Chordata species, the gasdermin sequences also presented homologous exons in the C-terminal domain (Additional file 1: Fig. S5C), suggesting that the repressor-cleavage-induced activation of gasdermin is a mechanism from the ancestral form.

### PJVK is a non-lytic member of the gasdermin family

**Fig. 6:**
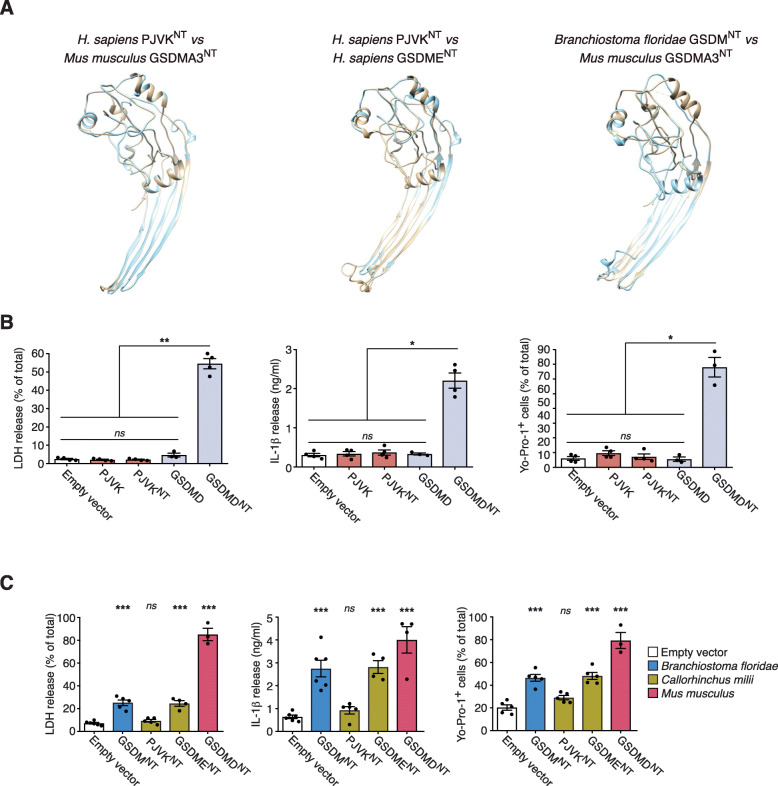
Full-length or N-terminal pejvakin expression does not induce pyroptosis. **A** Structural alignment of the molecular models of mouse GSDMA3^NT^ (6CB8) (brown) with the model for the template generated human PJVK^NT^ (cyan) (left panel). Structural alignment of human GSDME^NT^ (brown) with human PJVK^NT^ (Cyan) both generated from the mouse GSDMA3^NT^ template-based model (middle panel). Structural alignment of mouse GSDMA3^NT^ (6CB8) (brown) with the template-based model generated for jawless Chordate lancelet fish (*Branchiostoma floridae*) GSDM^NT^ (Cyan) (right panel). **B** Measurement of LDH release, IL-1β release, and Yo-Pro-1^+^ uptake in HEK293 cells that constitutively express the mature form of human IL-1β and transiently express human full length PJVK, PJVK^NT^, full length GSDMD, or GSDMD^NT^. **C** Measurement of LDH release, IL-1β release, and Yo-Pro-1^+^ uptake in HEK293 cells that constitutively express the mature form of human IL-1β and transiently express lancelet GSDM^NT^ (blue), shark PJVK^NT^, or GSDME^NT^ (dark yellow) or mouse GSDMD^NT^ (red). In **B** and **C,** each point represents an individual assay with at least *n*=3 to 6 different assays. *p<0.05, **p<0.01, and ***p<0.001; Kruskal-Wallis test with Dunn’s multiple comparison post-test

The lack of exons in the C-terminal domain in the p*jvk* gene suggested that the PJVK protein may be constitutively lytic. Indeed, structural analysis revealed that its N-terminal could adopt a lytic-like conformation (Fig. 6A). Human PJVK predicted structure was closer to hs-GSDME conformation, both in its full-length and N-terminal forms (Fig. 6A and Additional file 1: Fig. S6A). Additionally, we found that key residues important for binding the N-terminus to lipids and for the membrane insertion found in gasdermin (A-E) proteins were conserved in hs-PJVK (Fig. 5B). However, although the hs-PJVK N-terminal domain conserved 66% of the residues important for lipid binding and 47% of the residues important for membrane insertion, it only conserved 26% of residues important for homo-oligomerization when compared to the structure of the mm-GSDMA3 membrane pore [8]. Given that homo-oligomerization is essential for pore formation, this suggests that PJVK is a membrane protein which does not form pores, a finding that is congruent with the established role of PJVK in peroxisomes/peroxophagy [27].

The predicted lack of pore forming functionality of hs-PJVK was confirmed through ectopic expression of the full length of hs-PJVK or its N-terminal sequence up to the E^235^ residue in HEK293 cells. The N-terminus of PJVK was predicted up to the amino acid where the structural homology of the lytic N-terminal fragment finalizes when a structural homology was performed against the structure of the mm-GSDMA3 N-terminus. This did not induce loss of membrane integrity, loss of membrane pores, or cell death as was evidenced by measurements of Yo-Pro-1 uptake, LDH release, or IL-1β release (in stable HEK293 cells for IL-1β expression) (Fig. 6B and Additional file 1: Fig. S6B). Furthermore, the shark *Callorhinchus milii* (Chondrichthyes) PJVK^NT^ (sh-PJVK^NT^) (ELE^238^) failed to induce cell death (Fig. 6C and Additional file 1: Fig. S6B), suggesting that the loss of lytic functionality in PJVK is evolutionarily conserved.

### The ancestral proto-gasdermin was a pore-forming protein

The N-terminal domains of gasdermin proteins from distantly related organisms all had some pore-forming functionality. The N-terminal sequence from the Chordata *Branchiostoma floridae* gasdermin (KLE^251^) was able to adopt a structure similar to the one found for mouse GSDMA3 when forming a membrane pore (Fig. 6A). This structural similarity in gasdermins from distantly related organisms suggests functional conservation from the ancestral form. We provided further evidence for this using ectopic expression in HEK293 cells where *B. floridae* GSDM^NT^ (bf-GSDM^NT^), *C. milii* GSDME^NT^ (DLE^268^) (sh-GSDME^NT^), and *M. musculus* GSDMD^NT^ (LSD^276^) (mGSDMD^NT^) all induced the loss of membrane integrity and diffusion of soluble compounds into and out of the cell (Fig. 6C and Additional file 1: Fig. S6B). This finding and the conclusion that the ancestral proto-gasdermin was pore forming is further supported by the results reported for the recently described GSDM^NT^ of the coral *Orbicella faveolata* [36]. It is important to highlight that while bf-GSDM^NT^ and sh-GSDME^NT^ resulted in a similar Yo-Pro-1 uptake and IL-1β release when compared to mm-GSDMD^NT^, the release of LDH (a tetrameric protein) was significantly lower (Fig. 6C), suggesting that the ancestral proto-gasdermin function was primarily the permeabilization of the membrane and that the function of cell lysis evolved in gasdermin E after the divergence of Osteichthyes and Chondrichthyes.

### Expression of gasdermin family genes during infection

**Fig. 7.**
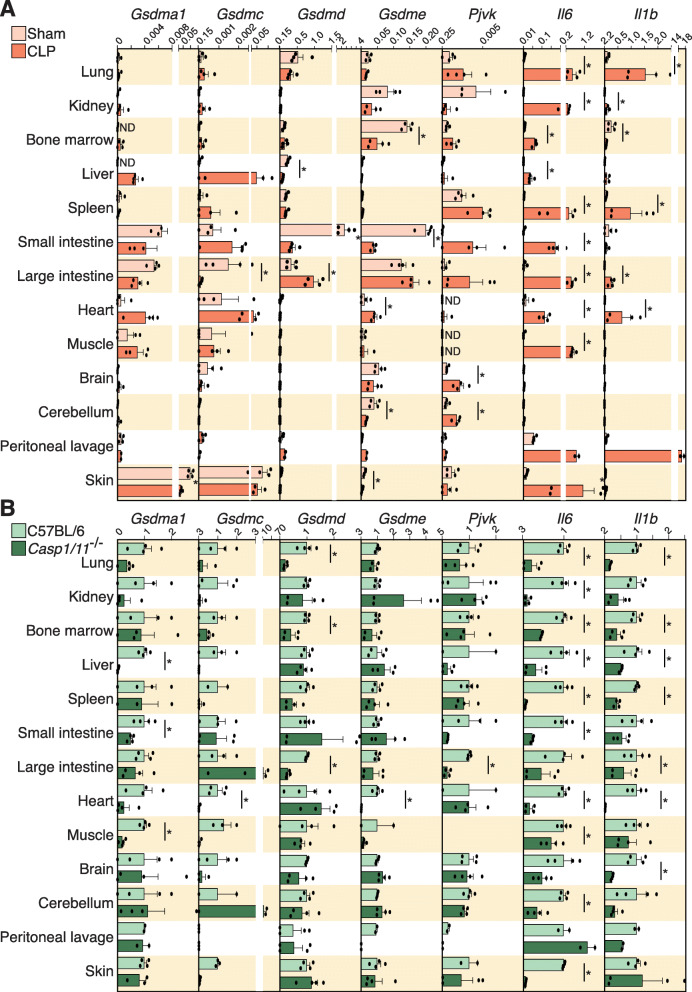
Gene expression of gasdermin genes in different mouse tissues during sepsis. **A** Relative expression of mouse *gsdma1*, *gsdmc*, *gsdmd*, *gsdme*, *pjvk*, *il6*, and *il1b* genes analyzed by quantitative PCR from different tissues of sham operated mice (yellow) and septic mice (CLP, in orange). **B** Fold change of the expression of mouse *gsdma1*, *gsdmc*, *gsdmd*, *gsdme*, *pjvk*, *il6*, and *il1b* genes analyzed by quantitative PCR from different tissues of septic wild type (C57BL/6, in light green) versus *Casp1/11*^-/-^ mice (dark green); *Casp1/11*^-/-^ expression was relative to wild type expression. Each point represents an independent sample from *n*= 2 mice. Mann-Whitney test for **A** and **B**, *p<0.05

Since pyroptotic cell death is an evolutionarily conserved function of gasdermins in response to infection [36], we next aimed to analyze the expression of the different gasdermins in different mouse tissues during a cecal ligation and puncture (CLP)-induced model of sepsis. Basal expression of mm-*gsdma1* and mm-*gsdmc* was found in the skin and intestines, mm-*gsdmd* expression was found in the lung and intestines, while basal mm-*gsdme* expression was found in the intestines, kidney, bone marrow, brain, and cerebellum (Fig. 7A). Mouse *pjvk* was primarily basally expressed in the kidney and spleen (Fig. 7A). After 24h of sepsis, mm-*gsdma1* and mm-*gsdmc* expression increased in the liver, heart, and muscle and decreased in large intestine (Fig. 7A). Mouse *gsdmd* expression increased in the large intestine while it decreased in the small intestine (Fig. 7A) and mm-*gsdme* decreased in all tissues, except for an increased expression in the heart (Fig. 7A). Interestingly, the same decrease in expression was found in mm-*gsdme* using the bone marrow-derived macrophages treated with the TLR4 agonist lipopolysaccharide or the TLR2 agonist Pam3-CSK4 as the proinflammatory stimuli (Additional file 1: Fig. S6C). Mouse *pjvk* expression was upregulated in different tissues, including the brain, cerebellum, and intestine (Fig. 7A). To confirm that inflammatory sepsis had been induced, we looked for and found that robust increases of the pro-inflammatory cytokines mm-*il1b* and mm-*il6* had occurred in almost all tissues examined (Fig. 7A). Since the immune response during sepsis is partly mediated by caspase-1 and -11 induced GSDMD-pyroptosis [37, 38], we used the *Casp1/11*^-/-^ mice to study gasdermin expression during sepsis. As expected, the expression of mm-*il1b* and mm-*il6* during sepsis was reduced in the *Casp1/11*^-/-^ mice (Fig. 7B). Similarly, the increase in gasdermin expression in different tissues during sepsis was also dependent on caspase-1 and -11 (Fig. 7B). In particular, in the *Casp1/11*^-/-^ mice we saw a downregulation of mm-*gsdma1* and mm-*gsdmc* in the liver, mm-*gsdmd* in the large intestine, mm-*gsdme* in the heart, and mm-*pjvk* in the intestine (Fig. 7B). These data confirm that caspase-1 and -11 control the expression of different inflammatory mediators during sepsis and also suggests the modulation of different pyroptotic routes in different tissues.

## Discussion

In this study, we have identified that gasdermins were present in early in metazoan evolution as a single gene. Then in the Vertebrata common ancestor, the gasdermin gene underwent a number of duplications which expanded the gasdermin family until it reached its maximum expansion in Mammalian organisms. Having multiple gasdermin proteins likely allowed for a greater diversity of pyroptotic cell death pathways and potential activation/regulation mechanisms in mammals. In fact, structural and functional analyses allow us to map the evolution of pore formation in mammals, lancelets, and sharks. Within the gasdermin family, *pjvk,* and *gsdme* were the result of the first gasdermin duplication event in Vertebrata, which likely occurred in the common ancestor of jawed vertebrates, with PJVK losing pore formation after this initial duplication. However, although the Bayesian analysis supports this duplication after divergence from invertebrate common sequence (in accordance with [29]), the maximum likelihood phylogenic analysis places *pjvk* sequences in the same node as the gasdermins from invertebrate organisms. However, this discrepancy may be resolved by our synteny analysis, which demonstrates that both *pjvk* and *gsdme* share a similar genomic environment that was drastically different from invertebrates.

Our study found that following the initial duplication event (i.e., the purifying selection pressure of *pjvk*) was reduced, thus allowing the accumulation of mutations. These mutations resulted in the loss of the exons coding for the C-terminal repressor domain which should have created a constitutively lytic protein; however, we found that the hs-PJVK lacked key residues identified in the mm-GSDMA3 as essential for oligomerization and thus pore formation [8]. We then confirmed this lack of pore-formation functionality in the PJVK of different species, including that of shark, using ectopic transcription models. These results are supported by a previous study where hs-PJVK^NT^ fused to GFP was not able to reduce cell viability [28]. This makes pejvakin the only gasdermin family member that does not have a role in pore formation; instead, recent reports show that mm-PJVK likely plays a role in peroxisome proliferation by regulating selective autophagic degradation [26, 27]. It remains to be determined if PJVK has an alternative activation pathway involving different posttranslational modifications, beyond protein cleavage, which results in it gaining lytic capabilities.

A previous study has been shown that once they have been activated, mammalian gasdermins A to E cause pyroptotic cell death [5]. In our study, we found that non-vertebrate Chordata gasdermins are more efficient at inducing plasma membrane permeabilization than inducing LDH release (an indicator of cell lysis). This places doubt on whether the ancestral gasdermin protein was a lytic protein. However, a recent study found that coral GSDM^NT^ is highly lytic and induces high levels of LDH release [36]. This suggests that ancestral Chordata gasdermins were less efficient at inducing cell death and that maybe the pores produced by them could be more efficiently excised from the plasma membrane by different mechanisms, including the endosomal sorting complexes required for transport machinery that has been shown to be able to remove GSDMD pores from the plasma membrane [39].

In invertebrates, the lytic function of the N-terminal sequence of gasdermins has been demonstrated for the coral gasdermin that is activated after caspase-3 cleavage during infection and that induces cell death [36]. In our study, we found that the N-terminal sequences of distantly related Chordata species (lancelet and shark) also induce a cell death characterized by plasma membrane permeability. However, while ancestral coral gasdermin was annotated as GSDME-like [36], we suggest naming it as a gasdermin because in invertebrates a single sequence of ortholog gasdermin is found and the phylogenetic tree does not classify these invertebrate sequences with any of the vertebrate gasdermins, including GSDME. Overall, pyroptosis is an early conserved mechanism of gasdermins that protects animals by controlling a rapid necrotic type of cell death.

Conserved analogous cell death programs arose early in bacteria and fungi, encoded by *rcd-1* gene, and were able to induce cell death when two distinct haplogroups of *rcd-1* co-expressed in the cell [31]. This is a starkly different activation mechanism compared to invertebrate and vertebrate gasdermins, where the excision of a repressor C-terminal domain by protein cleavage is required for activation [36]. These functional differences, the lack of any meaningful sequence identity and the fact that *rcd* genes are only found in fungi and bacteria [40] all suggest that *rcd* genes do not share a common ancestral gene with gasdermins and thus should not be considered to be gasdermin family members.

The basic structure of the teleost genome was established before the major diversification of teleost species. The common ancestor of the teleost clade underwent a whole genome duplication [34, 41]. Our research demonstrates that this event likely explains the presences of the two *gsdme* genes (*gsdmea* and *gsdmeb*) found in this clade, as there was strong conservation of genomic synteny around these two gene loci. It has been shown that the GSDMEa protein is processed by apoptotic caspases and therefore could be similar to the function of mammalian GSDME, and GSDMEb is processed by inflammatory caspases and could function similarly to mammalian GSDMD [42, 43]. However, as the Tetrapoda lineage diverged from the Teleostei ancestral lineage prior to the gene duplication event, these similarities represent parallel evolution.

The diversity of gasdermin in mammalian species suggests that pyroptosis pathways have expanded in mammals, and evolved to modulate cell death in different cells and tissues, where the right combination of gasdermin expression and activation of specific proteases occur. In this regard, human GSDMB protein has been found to be processed by granzyme A during cytotoxic T cell targeting [44], and a splice variant of human *gsdmb* that introduces an exon in the central linker sequence with a caspase-1 cleavage site induces pyroptosis after caspase-1 activation [35]. Caspase-3 induces apoptosis with a pyroptosis-like cellular process after cleavage of GSDME and this gasdermin is responsible for the late necrotic phenotype usually observed during apoptosis [14]. Caspase-1, as well as caspase-4/5/11 and caspase-8, neutrophil proteases and cathepsin are all able to process GSDMD in the linker sequence and induce pyroptosis [6, 9–13]. The canonical and non-canonical inflammasome activation leads to caspase-1 and caspase-4/5/11 activation respectively, with subsequent GSDMD processing as a key regulator mechanism of inflammation during sepsis [37, 38, 45, 46]. Here, we demonstrate that the lack of caspase-1/11 affects the expression of the different gasdermins in specific tissues during sepsis, indicating that tissue damage and subsequent organ dysfunction during sepsis could be mediated by the activation of several gasdermins.

## Conclusions

Overall, our study indicates that the ancestral Vertebrata gasdermin was present in the common ancestor of Metazoa as a pore forming protein activated through cleavage. The expansion of the gasdermin family in the common ancestor of jawed vertebrates allowed for a greater diversity of activation pathways and indeed functions, given the high variation that exists within the exons coding the linker sequence of the different gasdermins. The most singular member of the family is pejvakin, a product of the first expansion that lost its repressor domain and its pore-forming functionality in a range of organisms from sharks to mammals, whilst maintaining its membrane binding and insertion properties. The greatest expansion was seen in the common ancestor of mammals, in which duplications of the gasdermin A gene generated the gasdermin A subfamily. Although the gasdermin family diversified and grew over hundreds of millions of years of evolution, we nevertheless show that all family members likely retained their role in coordinating the physiological response to infection.

## Methods

### Sequence analysis

The different full sequences of the gasdermin family were found using the BLAST program on the National Center for Biotechnology Information server (blast.ncbi.nlm.nih.gov/Blast.cgi) [47], which was used to search for similarity with established full gasdermin sequences (Additional file 1: Table S1) or through performing BLAST/BLAT using the ensembl, ensembl metazoan, and ensembl fungi databases (www.ensembl.org, metazoa.ensembl.org, fungi.ensembl.org) [48], and the NCBI protein and nucleotide database (www.ncbi.nlm.nih.gov), using the sequences annotated with an asterisk in the Additional file 1: Table S1 as template sequences for the searches. The RCD-1 sequences are shown in Additional file 4. The ensembl databases were used to annotate the length for different introns and exons. The two sequences were directly compared using the Pairwise Sequence alignment Needle (emboss v6.0.1, RRID:SCR_007254) program, while multiple sequence alignment was carried out with the CLUSTALW2 program v1.2.4 (RRID:SCR_002909), both of which were from the European Bioinformatics Institute (www.ebi.ac.uk) [49, 50]. The phylogenetic trees were constructed on the basis of the amino acid sequence alignments with the CLUSTALX v2.1 program (RRID:SCR_017055) [51], using the neighbor-joining inference method [52] and Mega v11.0 to obtain nexus output format [53]. The Bayesian tree was performed using the Bayesian evolutionary analysis utility (BEAUti2 v2.6.4) as a graphic user interface tool for generating BEAST2 XML configuration file using Gamma distribution as amino acid substitution model [54]. The XML file generated was analyzed with BEAST2 software v2.6.4 (RRID:SCR_017307) for Bayesian evolutionary analysis using the BEAGLE library (RRID:SCR_001789) where Markov chains were run for 1,000,000 generations and sampled every 1000 [55]. TreeAnnotator v2.6.4 was used to create a maximum clade credibility tree with a burn-in of trees of 0 [56]. The maximum likelihood phylogeny tree was produced using the PhyML v3.0 portal (RRID:SCR_014629, http://www.atgc-montpellier.fr/phyml/) to obtain a maximum likelihood phylogeny tree based on the Akaike information criterion (AIC) with LG as substitution model, with a proportion of invariable sites fixed at 0.0, with an empirical equilibrium frequencies, with an estimated gamma shape parameter of 2.004, and a bootstrap of 100 [57]. Files used for Bayesian and maximum likelihood analysis are in Additional files 6 and 7. The different trees were displayed with FigTree v1.4.4 software (RRID:SCR_008515, tree.bio.ed.ac.uk/software/figtree) [58] or by using the Interactive Tree of Life v5.7 (iTOL, RRID:SCR_018174, https://itol.embl.de/itol.cgi) [59]. Synteny analysis was performed by searching the available genomes and using Softberry software (Fgenes v1.5, RRID:SCR_018928) (softberry.com) [60] to predict gene sequences within them. The accession numbers of all genes analyzed are indicated in the Additional file 1: Table S1.

### Protein homology modeling

The 3D model for the proteins of the GSDMs family was created using the Phyre2 Protein Fold Recognition Server v2.0 [61] (www.sbg.bio.ic.ac.uk/~phyre2) for the full length 3D models of human GSDME and PJVK (O60443, Q0ZLH3) The Cryo-EM structure of mouse Gasdermin A3 membrane pore (PDB: 6CB8) was used as template to determine the N terminal portion of the human PJVK^NT^, human GSDME^NT^, and lancelet GSDM^NT^ proteins. Full length and N terminal proteins were modeled with CHIMERA software v1.10.2 (UCSF ChimeraX, RRID:SCR_015872, www.cgl.ucsf.edu/chimera) [62].

### Plasmid constructs, cells, and transfections

The different GSDMs for human PJVK^FL^ (QOZLH3) and PJVK^NT^ (QOZLH3, amino acids 1–237), mouse GSDMD^NT^ (QQ9D8T2, amino acids 1–276), shark GSDME^NT^ (K4FYP8, amino acids 1–266), shark PJVK^NT^ (A0A4W3IS58, amino acids 1–236), and lancelet GSDM^NT^ (C3VN8, amino acids 1–249) were ordered from GenScript and cloned into the pcDNA3.1+N-MYC vector, except for human GSDMD-FLAG and human GSDMD^NT^-FLAG expression vectors that were kindly provided by F. Saho (National Institute of Biological Sciences, Beijing, China). HEK293T cells (CRL-11268; American Type Culture Collection) were maintained in DMEM:F12 (1:1) (Lonza, Verviers, Belgium) supplemented with 10% fetal calf serum (FCS) (Life Technologies), 2 mM Glutamax (Life Technologies), and 1% penicillin-streptomycin (Life Technologies). Cell line was not authenticated, but was free of mycoplasma by routinely testing with the MycoProbe Mycoplasma Detection Kit following manufacturer instructions (R&D Systems). Lipofectamine 2000 was used for the transfection of HEK293T cells according to the manufacturer’s instructions using the indicated amount of plasmid. HEK cells that constitutively express human IL-1β were used for ELISA assays, LDH release measurement and YO-PRO^TM^ uptake after plasmid transfection of the human PJVK^FL^ and human PJVK^NT^, mouse GSDMD^NT^, shark GSDME^NT^, shark PJVK^NT^, and lancelet GSDM^NT^ at the indicated times. All cells were routinely tested for mycoplasma contamination with a Mycoplasma Detection Kit (Roche). The bone marrow-derived macrophages (BMDMs) were obtained from wild-type mice using 25% L cell-conditioned media as previously described [63]. BMDMs were treated with *E. coli* LPS O55:B5 (Sigma-Aldrich) or Pam3-CSK4 (Invivogen) in their respective complete media in a final concentration of 1μg/ml.

### Western blot

HEK293T cells transfected with the different GSDM plasmids were lysed in ice-cold lysis buffer (50 mM Tris-HCl pH8.0, 150 mM NaCl, 2% Triton X-100, supplemented with 100 μl/ml of protease inhibitor mixture (Sigma) for 30 min on ice) and were then clarified by centrifugation (16,000 g for 15 min at 4°C). Cell lysates were resolved in 4–12% precast Criterion polyacrylamide gels (Biorad) and transferred to nitrocellulose membranes (BioRad) by electroblotting as described in a previous study [64]. The membranes were probed with anti-MYC mouse monoclonal (clone 4A6, EMD Milipore Cat# 05-724, RRID:AB_309938, 1:1000), anti-FLAG mouse monoclonal (clone M2, Sigma, Cat# F1804, RRID:AB_262044, 1:1000), and horseradish peroxidase-anti-β-actin (clone C4, Santa Cruz, Cat# sc-47778 HRP, RRID:AB_2714189, 1:10000). Antibody validation was performed against cell lysates of un-transfected HEK293T cells without expression of tagged GSDMs. Uncropped Western blot can be found in Additional file 8.

### Lactate dehydrogenase (LDH) release and Yo-Pro-1 uptake assays

LDH release was measured using the Cytotoxicity Detection kit (Roche, Barcelona, Spain) following the manufacturer’s instructions and expressed as percentage of total cell LDH content. For Yo-Pro uptake, HEK293T cells transfected with the different GSDMs plasmids were incubated with 2.5 μM Yo-Pro-1 for 5 min and measured at 485±9/515±9 nm with bottom excitation/emission in the Synergy Mx plate reader (BioTek); the percentage of YO-PRO^TM^ positive cells is shown in the results.

### ELISA assay

Human IL-1β released to the cell supernatant was measured using ELISA from Thermo Fisher Scientific following the manufacturer’s instructions and then read in a Synergy Mx (BioTek) plate reader.

### Cecal ligation and puncture model

Cecal ligation and puncture-induced sepsis were performed as previously described [64, 65] in C57BL/6 (WT, wild-type, IMSR Cat# JAX:000664, RRID:IMSR_JAX:000664) and caspase-1/11 deficient (*Casp1/11*^*-*/-^) (MGI Cat# 2179469, RRID:MGI:2179469) mice in C57BL/6 background. For all experiments, mice between 8 and 10 weeks of age were used. Mice were bred in specific pathogen-free conditions with a 12:12-h light-dark cycle. Laparotomy was performed to isolate the cecum of mice anesthetized with isoflurane. Approximately 2/3 of the cecum was ligated with a 6-0 silk suture and punctured twice through-and-through with a 21-gauge needle. The abdominal wall and incision were then closed with 6-0 silk suture. Sham-operated animals underwent laparotomy without ligation or puncture of the cecum. Buprenorphine (0.3 mg/kg) was administered intraperitoneally at the time of surgery, and mice were monitored continuously until recovery from anesthesia. For sample collection, 24 h after the procedure, animals were euthanized with CO_2_ inhalation and peritoneal lavages were performed with 4 ml of sterile saline and cells were centrifuged. For tissue harvesting, the abdominal wall was exposed and the organs were removed using scissors and forceps prior to storage at −80°C for future analysis.

### Quantitative reverse transcriptase-PCR analysis

Detailed methods used for qRT-PCR have been described previously [63]. In brief, the total RNA was extracted from tissues and organs of wild type and *Casp1/11*^-/-^ mice using QIAazol lysis reagent (Qiagen) following the manufacturer’s instructions. The RNA was then purified using the RNeasy kit (Qiagen) according to the manufacturer’s recommendations and quantified on a NanoDrop 2000 (Thermo Fisher Scientific). Reverse transcription was carried out using iScript TM cDNA Synthesis kit (BioRad). qPCR was performed using an iQTM 5 Real-Time PCR (BioRad) with a SYBR Green mix (Takara), and the primers used were obtained from Sigma-Aldrich (KiCqStart Primers, Additional file 5). Relative gene expression levels were calculated using the 2^-ΔCt^ method normalizing to *Hprt1* expression as endogenous control. The foldchange expression was calculated using the 2^-ΔΔCt^ method.

### Statistical analysis

In all of the statistical analyses shown in the figures, the data represent the mean ± standard error of the mean. The data were analyzed by non-parametric unpaired Mann-Whitney two-tailed *U* test to determine the differences between two conditions. When more than two conditions were considered, the non-parametric Kruskal-Wallis test with Dunn’s multiple comparisons post-test were used. In all cases, Prism’s Graph Pad software was used for data calculation and representation. In the figure legends, the asterisks denote statistically significant differences between the treatments (*p<0.05, **p<0.005, and ***p<0.001).

## Supplementary Information

**Additional file 1: Figures S1-S6; Table S1**.

**Additional file 2:** Figure 6 source data.

**Additional file 3:** Figure 7 source data.

**Additional file 4:** Fungal regulated cell death (RCD) sequences used in this study.

**Additional file 5:** Primer sequences data.

**Additional file 6:** File source data for for Bayesian analysis.

**Additional file 7:** File source data for Maximum likelihood analysis.

**Additional file 8:** Uncropped Western blots.

## Data Availability

The datasets supporting the conclusions of this article are included within the article and its additional files.

## Abbreviations

BMDM: Bone marrow-derived macrophages
CLP: Cecal ligation and puncture
FCS: Fetal calf serum
GSDM: Gasdermin
HEK293: Human embryonic kidney 293 cells
HOX: Homeobox genes
IL: Interleukin
LDH: Lactate dehydrogenase
NET: Neutrophil-derived extracellular trap
PJVK: Pejvakin
RCD-1: Regulated cell death-1
WT: Wild-type
