# Supplementary material for: Evolutionary analyses of the gasdermin family suggest conserved roles in infection response despite loss of pore-forming functionality
Source: BMC Biol. 2022 Jan 7;20:9. doi: 10.1186/s12915-021-01220-z (PMC8742441; doi:10.1186/s12915-021-01220-z)
Supplement: Supplementary file 1 — Additional file 1: Figures S1-S6; Table S1. [file 12915_2021_1220_MOESM1_ESM.pdf]

Additional file 1:

Table S1. Accession number of the different genes used in this study. Asterisks denotes the sequences used as templates in *ensembl* and NCBI databases.

Filo/Class	Specie	Common name	Gene	Accession number
Chordata/ Mammalia	*Homo sapiens*	Human	*gsdma* *	Q96QA5
			gsdmb *	Q8TAX9
			gsdmc *	Q9BYG8
			gsdmd *	P57764
			gsdme *	O60443
			pjvk *	Q0ZLH3
	Mus musculus	Mouse	*gsdma1*	Q9EST1
			gsdma2	Q32M21
			gsdma3	Q5Y4Y6
			gsdmc1	Q99NB5
			gsdmc2	Q2KHK6
			gsdmc3	Q8CB12
			gsdmc4	Q3TR54
			gsdmd	Q9D8T2
			gsdme	Q9Z2D3
			pjvk	Q0ZLH2
	Bos taurus	Cow	*gsdma*	F1MUE4
			gsdmb	F1MCQ4
			gsdmc	Q2KJ26
			gsdmd	Q29S10
			gsdme	E1BFC3
			pjvk	E1BK64
	Sus scrofa	Pig	*gsdma*	F1RXA6
			gsdmb	I3LUA2
			gsdmc	F1RRS0
			gsdmd	F1SEV7
			gsdme	F1ST94
			pjvk	F1RZD1
	Gorilla gorilla gorilla	Gorilla	*gsdma*	G3QQR5
			gsdmb	G3QZ02
			gsdmc	G3QNY2
			gsdmd	A0A2I2ZBS6
			gsdme	G3QR26
			pjvk	G3R652
	Neomonachus schauinslandi	Seal	*gsdmax1*	XP_021560075
			gsdmax2	XP_021560077
			gsdmb	XP_021560045
			gsdmd	XP_021544405
			gsdme	XP_021558921
			pjvk	XP_021545343
	Orcinus orca	Whale	*gsdmb*	XP_033275830
			gsdmc	XP_033266659
			gsdme	XP_004265687
			pjvk	XP_033279542

Chordata/ Aves	*Ficedula albicollis*	Flycatcher	*gsdma*<br>*gsdme*<br>*pjvk*	U3JCH6<br>U3K4L7<br>U3JL98
	Meleagris gallopavo	Turkey	*gsdma*<br>*gsdme*<br>*pjvk*	H9H0H2<br>G3UR30<br>G1NB24
	Gallus gallus	Chicken	*gsdma*<br>*gsdme*<br>*pjvk*	H9L060<br>F1NP12<br>E1BYV7
Chordata/ Reptilia	*Anolis carolinensis*	Lizard	*gsdma*<br>*gsdme*<br>*pjvk*	G1KWU3<br>G1KNI3<br>H9GP49
	Pelodiscus sinensis	Turtle	*gsdma*<br>*gsdme*<br>*pjvk*	K7FU99<br>K7GG46<br>K7F4E5
Chordata/ Amphibian	*Xenopus Tropicalis*	African frog	*gsdme*<br>*pjvk*	A0A7D9NJQ2<br>A0A6I8SVR2
Chordata/ Sarcopterygii	*Latimeria chalumnae*	Coelacant	*gsdme*<br>*pjvk*	H3AEY8<br>H3A539
Chordata/ Osteychyes	*Oryzias laties*	Medaka	*gsdmea*<br>*gsdmeb*<br>*pjvk*	H2LKI2<br>H2LUH2<br>H2L2X9
	Tetraodon nigroviridis	Tetraodon	*gsdmea*<br>*gsdmeb*<br>*pjvk*	H3C1H7<br>H3C9T5<br>H3CZ40
	Danio rerio	Zebra fish	*gsdmea*<br>*gsdmeb*<br>*pjvk* *	E7F9U2<br>Q6J2R6<br>F8W4T7
	Takifugu rubripes	Japanese puffer	*gsdmea*<br>*gsdmeb*<br>*pjvk*	A0A3B5K424<br>H2UMH9<br>H2U9V8
	Oreochromis niloticus	Tilapia	*gsdmeb*<br>*pjvk*	I3J4F1<br>I3JJG0
Chordata/ Chondrichthyes	*Callorhinchus milii*	Elephant shark	*gsdme*<br>*pjvk*	K4FYP8<br>A0A4W3IS58
Chordata/ Hyperoartia	*Petromyzon Marinus*	Lamprey	*gsdm*	S4RLH7
Chordata/ Leptocardii	*Branchiostoma floridae*	Florida lancelet	*gsdm*	C3ZVN8
Non-Chordata/ Cnidaria	*Nematostella vectensis*	Anemone	*gsdm* *	A7T146
	Exaiptasi pallida	Anemone	*gsdm*	XP_020910515.1
	Orbicella faveolata	Coral	*gsdm*	XP_020607257.1
	Stylophora pistillata	Coral	*gsdm*	XP_022788280.1
	Acropora digitifera	Coral	*gsdm*	XP_015769608.1
	Hydra vulgaris	Fresh water polyp	*gsdm*	XP_012557585.1
Non-Chordata/ Brachiopoda	*Lingula anatina*	Marine worm	*gsdm*	A0A1S3HNQ5
	Saccoglossus kowalevskii	Marine worm	*gsdm*	XP_006824139.1
	Lottia gigantea	Sea snail	*gsdm*	V4CLM6

Non-Chordata/ Mollusca	*Pomacea canaliculata*	Sea snail	*gsdm*	XP_025094681
	Octopus bimaculoides	Octopus	*gsdm*	A0A0L8I419
Non-chordata/ Echinodermata	*Strongylocentrotus purpuratus*	Purple sea urchin	*gsdm1*<br>*gsdm2*	W4ZF48<br>XP_030830372.1
Non-chordata/ Placozoa	*Trichoplax adhaerens*		*gsdm*	B3RMM6

Figure S1

A

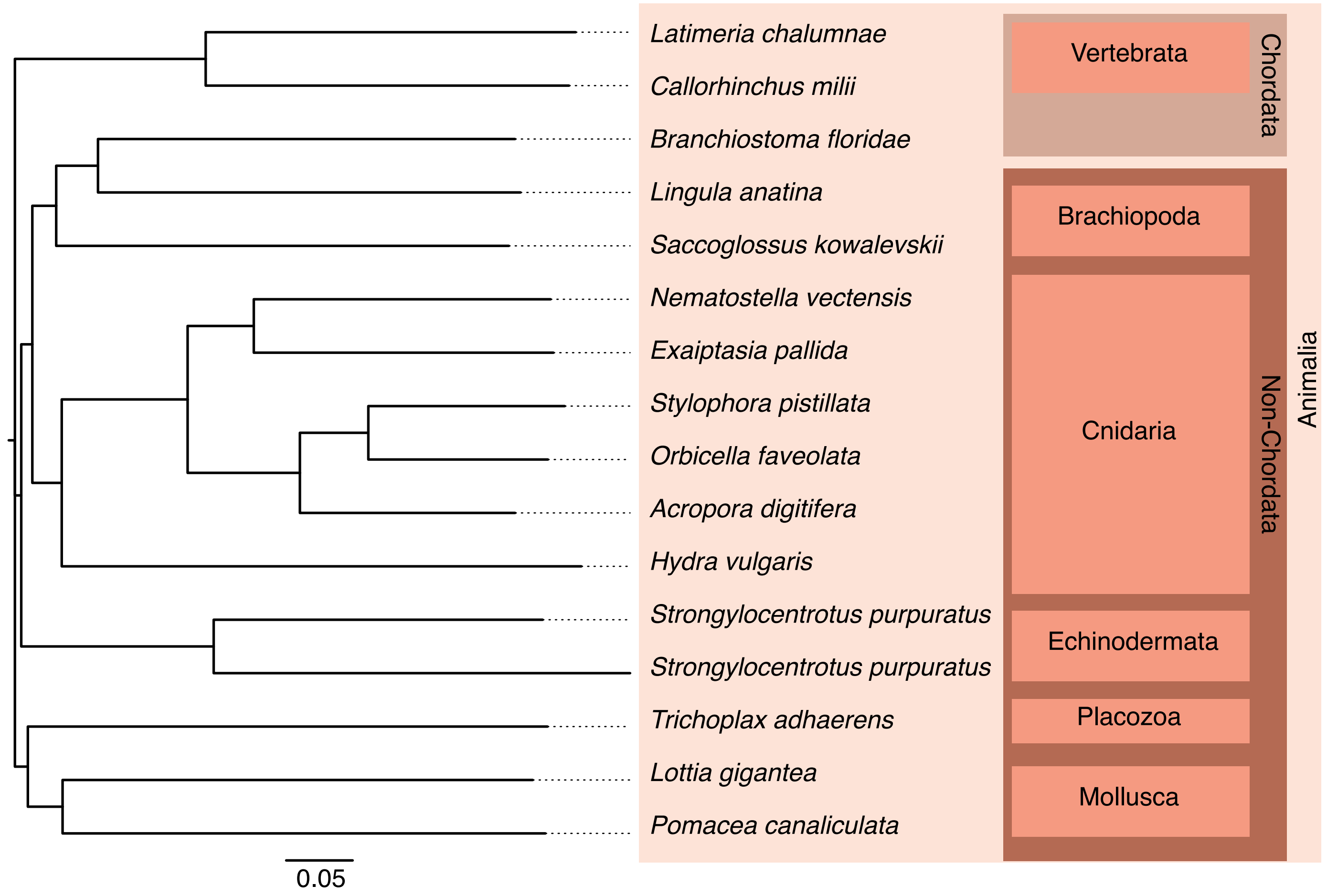

Latimeria chalumnae
Callorhinchus milii
Branchiostoma floridae
Lingula anatina
Saccoglossus kowalevskii
Nematostella vectensis
Exaiptasia pallida
Stylophora pistillata
Orbicella faveolata
Acropora digitifera
Hydra vulgaris
Strongylocentrotus purpuratus
Strongylocentrotus purpuratus
Trichoplax adhaerens
Lottia gigantea
Pomacea canaliculata
0.05
Vertebrata
Chordata
Brachiopoda
Cnidaria
Echinodermata
Placozoa
Mollusca
Non-Chordata
Animalia

B

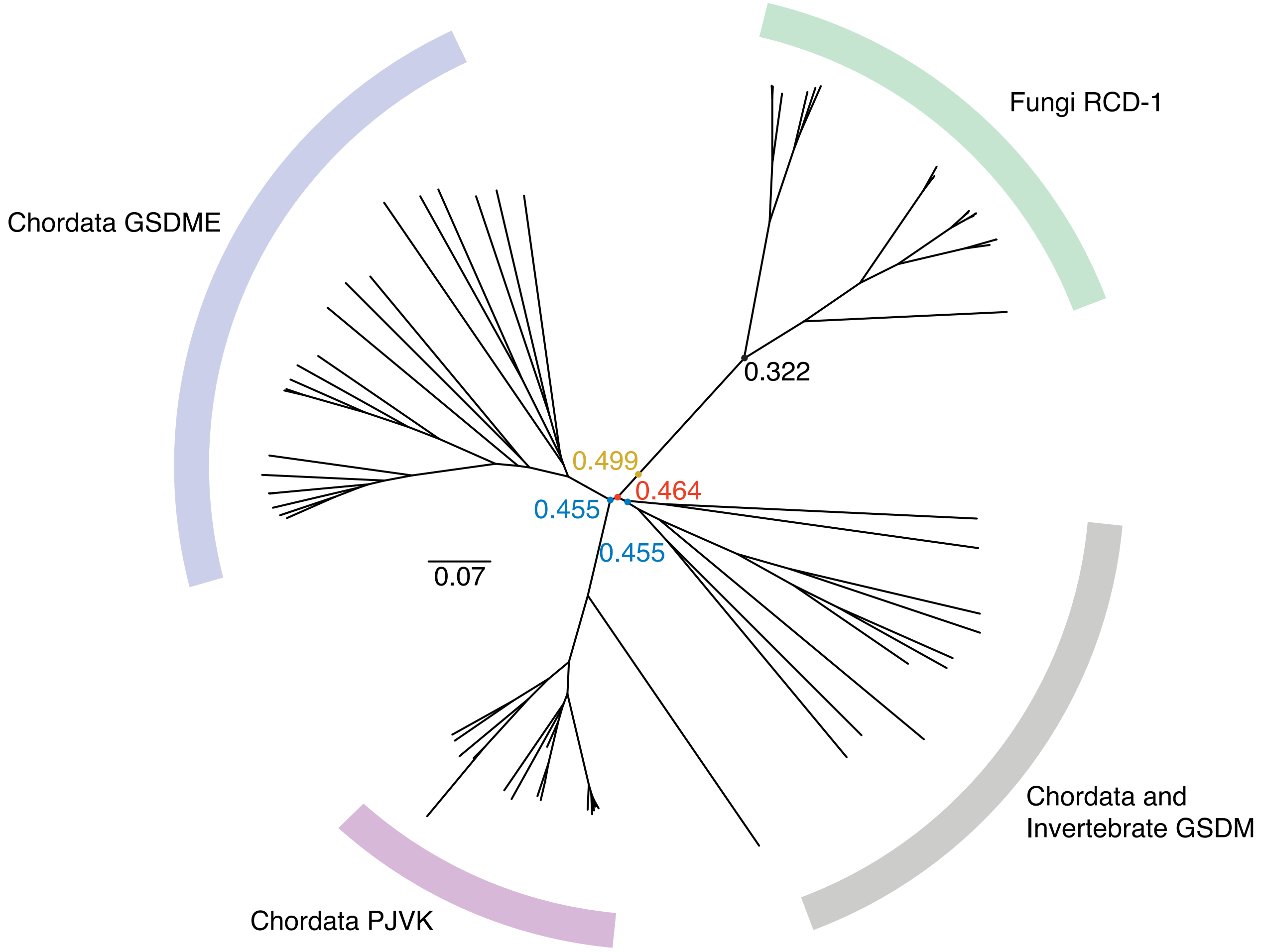

Chordata GSDME
Fungi RCD-1
0.322
0.499
0.464
0.455
0.455
0.07
Chordata and Invertebrate GSDM
Chordata PJVK

Figure S1. Phylogeny of the gasdermin gene family in invertebrates. (A) Phylogenetic tree inferred from amino acid sequence data using the neighbor-joining method for the different gasdermin sequences found in invertebrates. Chordata *gsdme* sequences of coelacanth (*Latimeria chalumnae*) and shark (*Callorhinchus milii*) were also included in the analysis. (**B**) Phylogenetic tree inferred from amino acid sequence data using the neighbor-joining method for the different gasdermin sequences and fungal RCD-1 sequences as annotated.

Figure S2

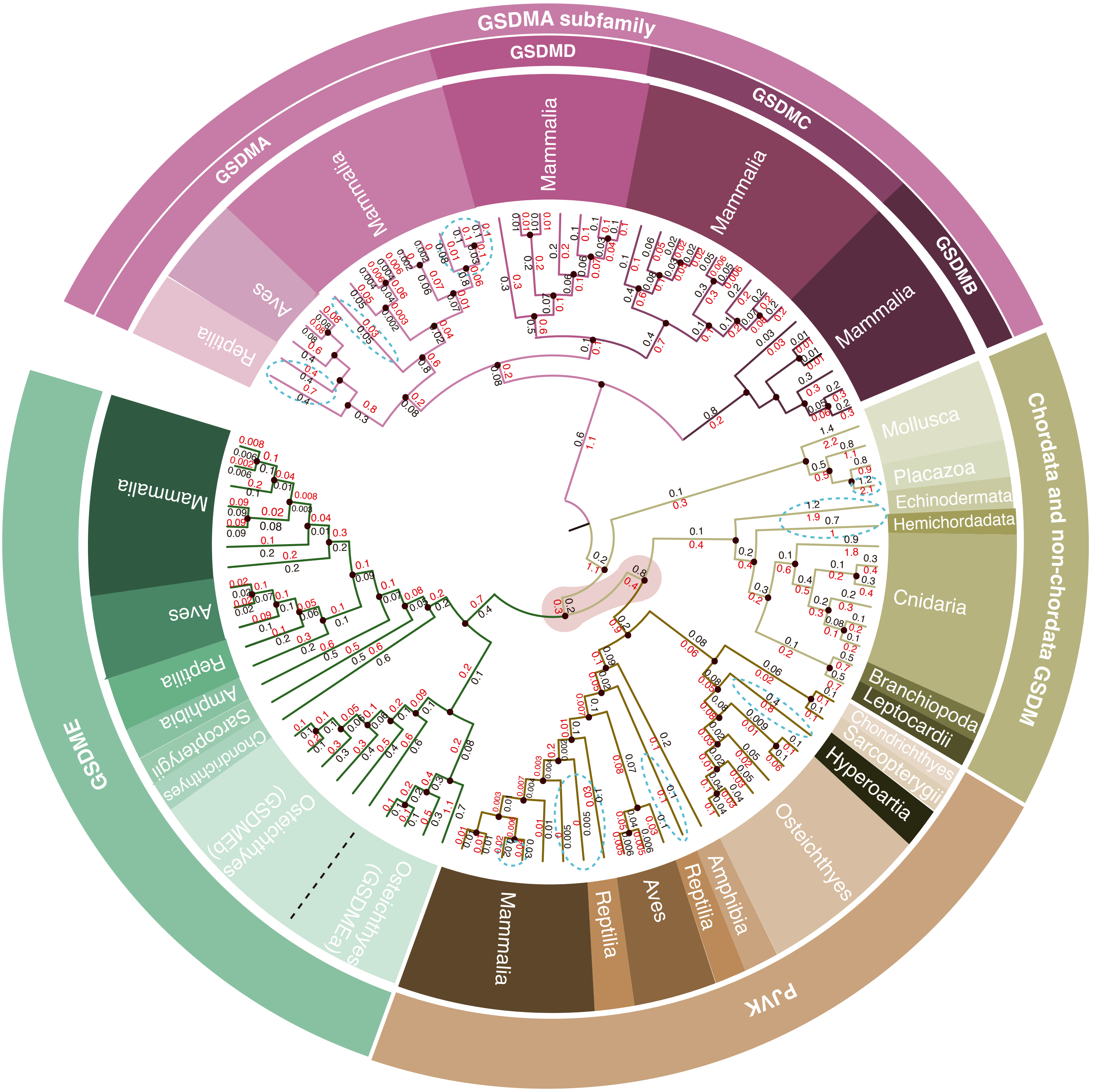
GSDMA subfamily
GSDMA
GSDMD
GSDMC
GSDMB
Mammalia
Aves
Reptilia
Chordata and non-chordata GSDM
Mollusca
Placazoa
Echinodermata
Hemichordadata
Cnidaria
Branchiopoda
Leptocardii
Chondrichthyes
Sarcopterygii
Hyperoartia
Osteichthyes
Amphibia
PJVK
GSDME
Osteichthyes (GSDMEa)
Osteichthyes (GSDMEb)

Figure S2. Phylogeny of the gasdermin family. Phylogenetic tree inferred from amino acid sequence data using the maximum likelihood method showing divergence of full-length gasdermins in mammals, birds, reptiles, amphibians, fish, jawless Chordata and non-Chordata. Black numbers represent relative branch length of Bayesian analysis and red numbers relative branch length of maximum likelihood. Light blue dashed circles show small changes between the Bayesian and maximum likelihood methods. Pink circle show how maximum likelihood analysis place PJVK sequences in the same node as the gasdermins from invertebrates and gasdermin E in a different node, which is the main difference when compared to the Bayesian phylogenetic tree.

Figure S3

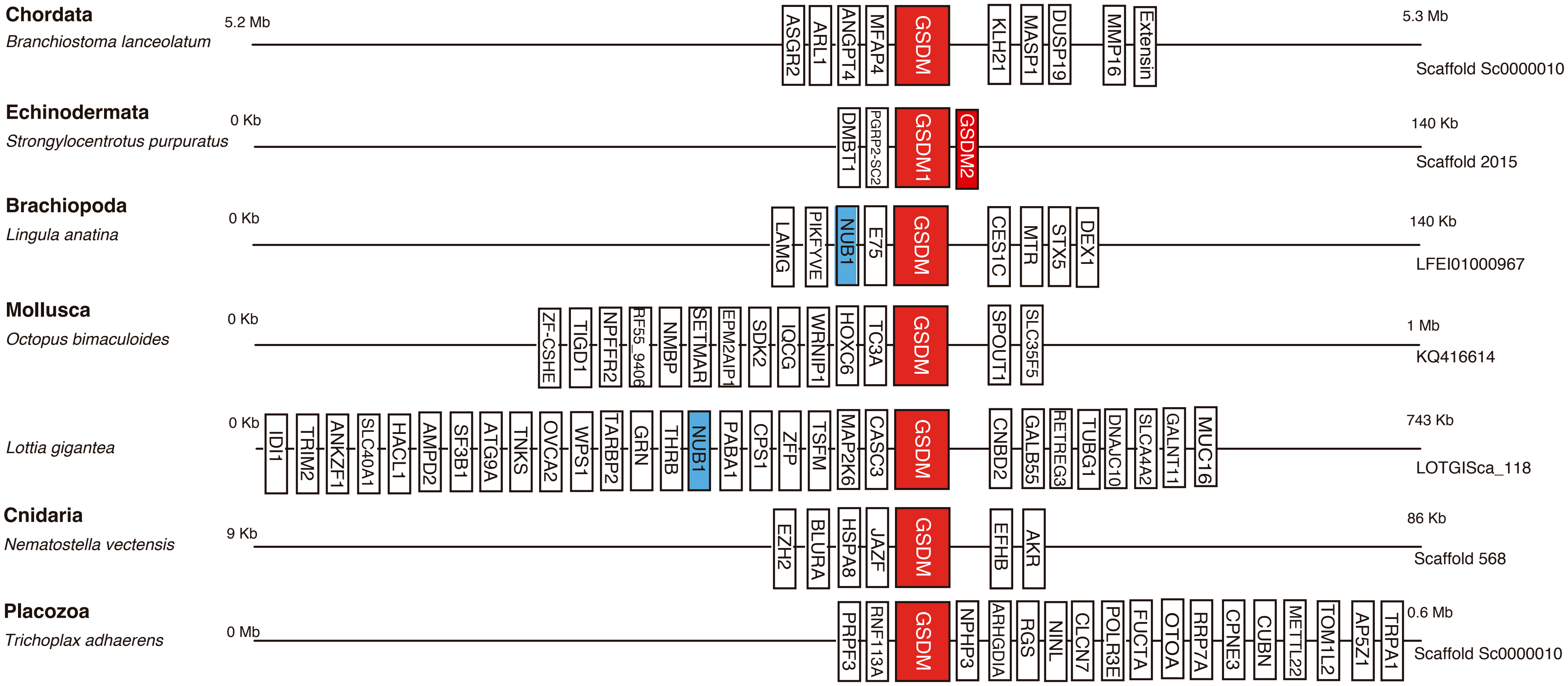
Chordata
Branchiostoma lanceolatum
5.2 Mb
ASGR2
ARL1
ANGPT4
MFAP4
GSDM
KLH21
MASP1
DUSP19
MMP16
Extensin
5.3 Mb
Scaffold Sc0000010
Echinodermata
Strongylocentrotus purpuratus
0 Kb
DMBT1
PGRP2-SC2
GSDM1
GSDM2
140 Kb
Scaffold 2015
Brachiopoda
Lingula anatina
0 Kb
LAMG
PIKFYVE
NUB1
E75
GSDM
CES1C
MTR
STX5
DEX1
140 Kb
LFEI01000967
Mollusca
Octopus bimaculoides
0 Kb
ZF-CSHE
TIGD1
NPFFR2
RF55_9406
NMBP
SETMAR
EPM2AIP1
SDK2
IQCG
WRNIP1
HOXC6
TC3A
GSDM
SPOUT1
SLC35F5
1 Mb
KQ416614
Lottia gigantea
0 Kb
IDI1
TRIM2
ANKZF1
SLC40A1
HACL1
AMPD2
SF3B1
ATG9A
TNKS
OVCA2
WPS1
TARBP2
GRN
THRB
NUB1
PABA1
CPS1
ZFP
TSFM
MAP2K6
CASC3
GSDM
CNBD2
GALB55
RETREG3
TUBG1
DNAJC10
SLCA4A2
GALNT11
MUC16
743 Kb
LOTGISca_118
Cnidaria
Nematostella vectensis
9 Kb
EZH2
BLURA
HSPA8
JAZF
GSDM
EFHB
AKR
86 Kb
Scaffold 568
Placozoa
Trichoplax adhaerens
0 Mb
PRPF3
RNF113A
GSDM
NPHP3
ARHGDIA
RGS
NINL
CLCN7
POLR3E
FUCTA
OTOA
RRP7A
CPNE3
CUBN
METTL22
TOM1L2
AP5Z1
TRPA1
0.6 Mb
Scaffold Sc0000010

Figure S3. Synteny analysis of the gasdermin loci in early- and non-Chordata species. Schematic diagram showing the conservation of synteny in the *gsdm* loci of different early Chordata (*Branchiostoma lanceolatum*) and non-Chordata species (*Stongylocentrotus purpuratus, Lingula anatina, Octopus bimaculoides, Lottia gigantea, Nematostella vectensis* and *Trichoplax adhaerens*).

Figure S4

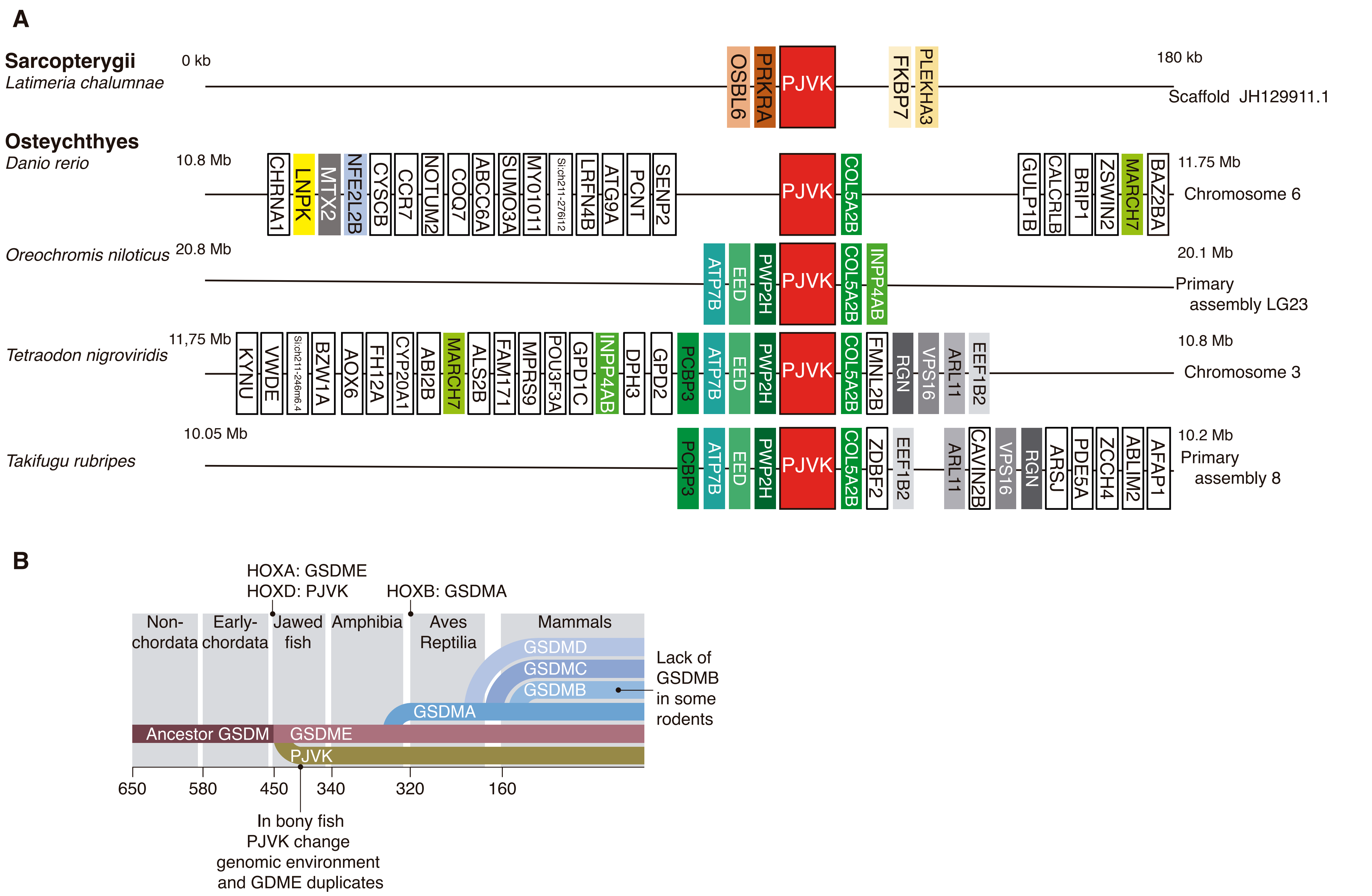

A
Sarcopterygii
Latimeria chalumnae
0 kb
180 kb
Scaffold JH129911.1
OSBL6
PRKRA
PJVK
FKBP7
PLEKHA3
Osteychthyes
Danio rerio
10.8 Mb
11.75 Mb
Chromosome 6
CHRNA1
LNPK
MTX2
NFE2L2B
CYSCB
CCR7
NOTUM2
COQ7
ABCC6A
SUMO3A
MYO1011
Si:ch211-276i12
LRFN4B
ATG9A
PCNT
SENP2
PJVK
COL5A2B
GULP1B
CALCRLB
BRIP1
ZSWIN2
MARCH7
BAZ2BA
Oreochromis niloticus
20.8 Mb
20.1 Mb
Primary assembly LG23
ATP7B
EED
PWP2H
PJVK
COL5A2B
INPP4AB
Tetraodon nigroviridis
11,75 Mb
10.8 Mb
Chromosome 3
KYNU
VWDE
Si:ch211-246m6.4
BZW1A
AOX6
FH12A
CYP20A1
ABI2B
MARCH7
ALS2B
FAM171
MPRS9
POU3F3A
GPD1C
INPP4AB
DPH3
GPD2
PCBP3
ATP7B
EED
PWP2H
PJVK
COL5A2B
FMNL2B
RGN
VPS16
ARL11
EEF1B2
Takifugu rubripes
10.05 Mb
10.2 Mb
Primary assembly 8
PCBP3
ATP7B
EED
PWP2H
PJVK
COL5A2B
ZDBF2
EEF1B2
ARL11
CAVIN2B
VPS16
RGN
ARSJ
PDE5A
ZCCH4
ABLIM2
AFAP1
B
HOXA: GSDME
HOXD: PJVK
HOXB: GSDMA
Non-chordata
Early-chordata
Jawed fish
Amphibia
Aves Reptilia
Mammals
GSDMD
GSDMC
GSDMB
GSDMA
Ancestor GSDM
GSDME
PJVK
Lack of GSDMB in some rodents
650
580
450
340
320
160
In bony fish PJVK change genomic environment and GDME duplicates

Figure S4. Synteny analysis of PJVK loci in fish. (**A**) Schematic diagram showing the conservation of synteny in the *pjvk* loci of different fish, including coelacanth (*Latimeria chalumnae*), zebra fish (*Danio rerio*), tilapia (*Oreochromis niloticus*), tetraodon (*Tetraodon nigroviridis*) and fugu (*Takifugu rubripes*). (**B**) Schematic representation of the evolution of gasdermins.

Figure S5

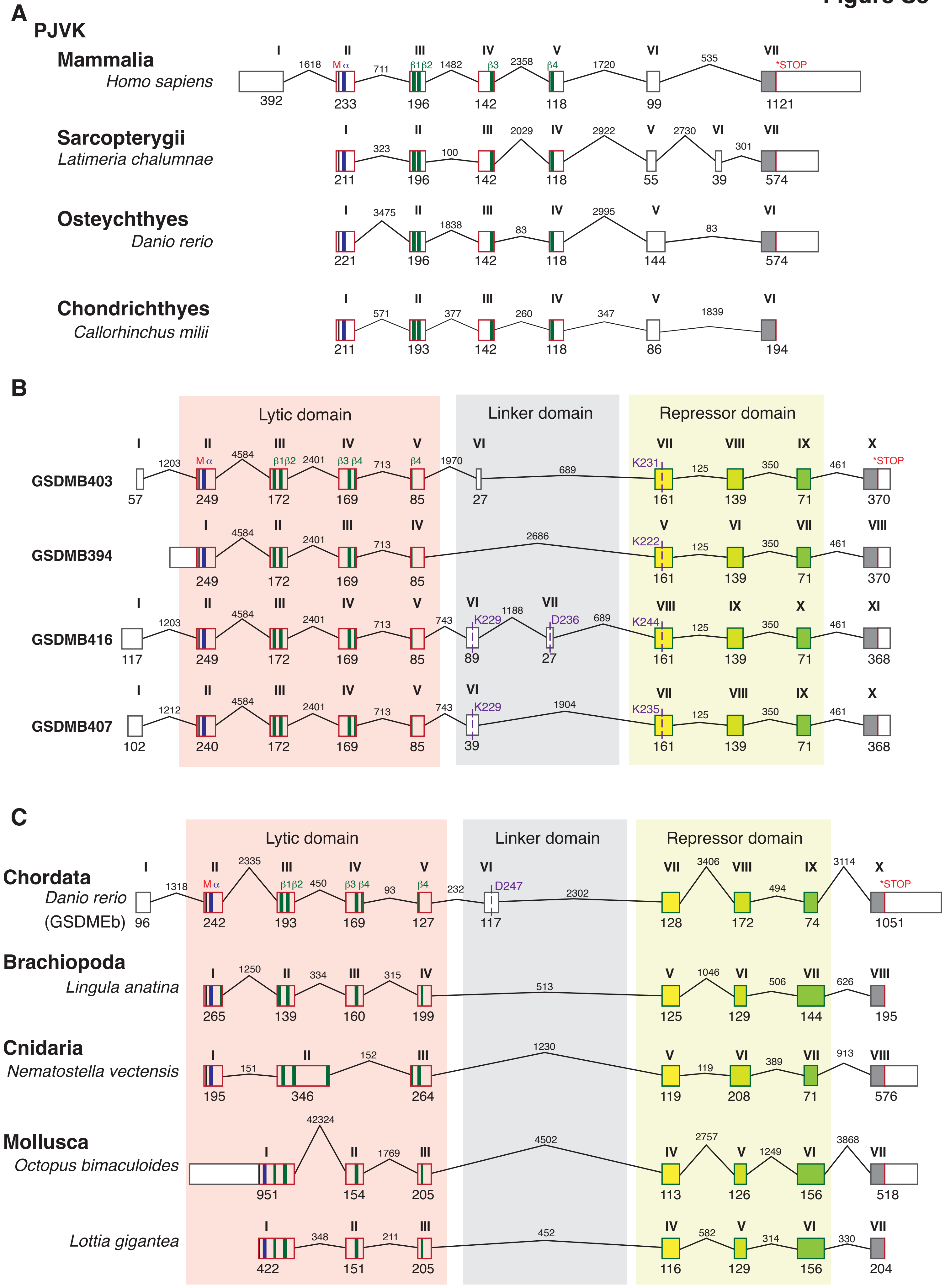
A
PJVK
Mammalia
Homo sapiens
I II III IV V VI VII
1618 711 1482 2358 1720 535
M α β1β2 β3 β4 *STOP
392 233 196 142 118 99 1121
Sarcopterygii
Latimeria chalumnae
I II III IV V VI VII
323 100 2029 2922 2730 301
211 196 142 118 55 39 574
Osteychthyes
Danio rerio
I II III IV V VI
3475 1838 83 2995 83
221 196 142 118 144 574
Chondrichthyes
Callorhinchus milii
I II III IV V VI
571 377 260 347 1839
211 193 142 118 86 194
B
Lytic domain
Linker domain
Repressor domain
GSDMB403
I II III IV V VI VII VIII IX X
1203 4584 2401 713 1970 689 125 350 461
M α β1β2 β3 β4 β4 K231 *STOP
57 249 172 169 85 27 161 139 71 370
GSDMB394
I II III IV V VI VII VIII
4584 2401 713 2686 125 350 461
K222
249 172 169 85 161 139 71 370
GSDMB416
I II III IV V VI VII VIII IX X XI
1203 4584 2401 713 743 1188 689 125 350 461
K229 D236 K244
117 249 172 169 85 89 27 161 139 71 368
GSDMB407
I II III IV V VI VII VIII IX X
1212 4584 2401 713 743 1904 125 350 461
K229 K235
102 240 172 169 85 39 161 139 71 368
C
Lytic domain
Linker domain
Repressor domain
Chordata
Danio rerio
(GSDMEb)
I II III IV V VI VII VIII IX X
1318 2335 450 93 232 2302 3406 494 3114
M α β1β2 β3 β4 β4 D247 *STOP
96 242 193 169 127 117 128 172 74 1051
Brachiopoda
Lingula anatina
I II III IV V VI VII VIII
1250 334 315 513 1046 506 626
265 139 160 199 125 129 144 195
Cnidaria
Nematostella vectensis
I II III V VI VII VIII
151 152 1230 119 389 913
195 346 264 119 208 71 576
Mollusca
Octopus bimaculoides
I II III IV V VI VII
42324 1769 4502 2757 1249 3868
951 154 205 113 126 156 518
Lottia gigantea
I II III IV V VI VII
348 211 452 582 314 330
422 151 205 116 129 156 204

Figure S5. Analysis of the exons and introns of different gasdermin genes. (**A**) Schematic representation of the different exons and introns present in the genomic DNA of *pjvk* found in human (*Homo sapiens*), coelacanth (*Latimeria chalumnae*), zebra fish (*Danio rerio*) and shark (*Callorhinchus milii*). (**B**) Schematic representation of the different exons and introns of the different human GSDMB splice variants; the granzyme A cleavage site at K^{229}, K^{231}, K^{222} and K^{235} and the caspase-1 cleavage site (D^{236}) are shown. (**C**) Schematic representation of the different exons and introns found in zebra fish (*Danio rerio*) *gsdmeb* and in non-Chordata gasdermin from Brachiopoda (*Lingula anatina*), Cnidaria (*Nematostella vectensis*) and Mollusca (*Octopus bimaculoides* and *Lottia gigantea*). In **A**, **B** and **C** the first methionine is indicated with a black line, the stop codon with a red line, the first α-helix of the N-terminal domain is indicated with a blue bar and the four β-sheets are indicated with green bars. The exons of the repressor C-terminal domain are represented with different colors to show the conserved similarity between them.

Figure S6

A

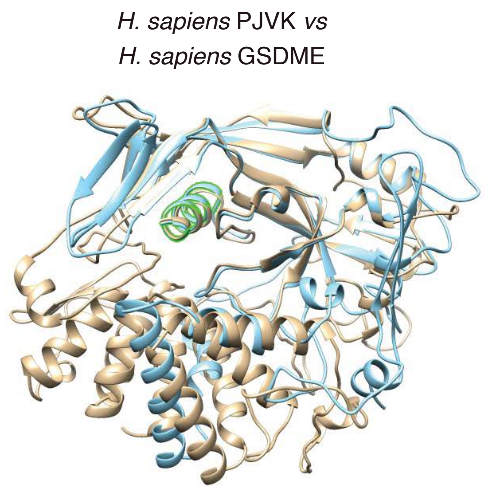

B

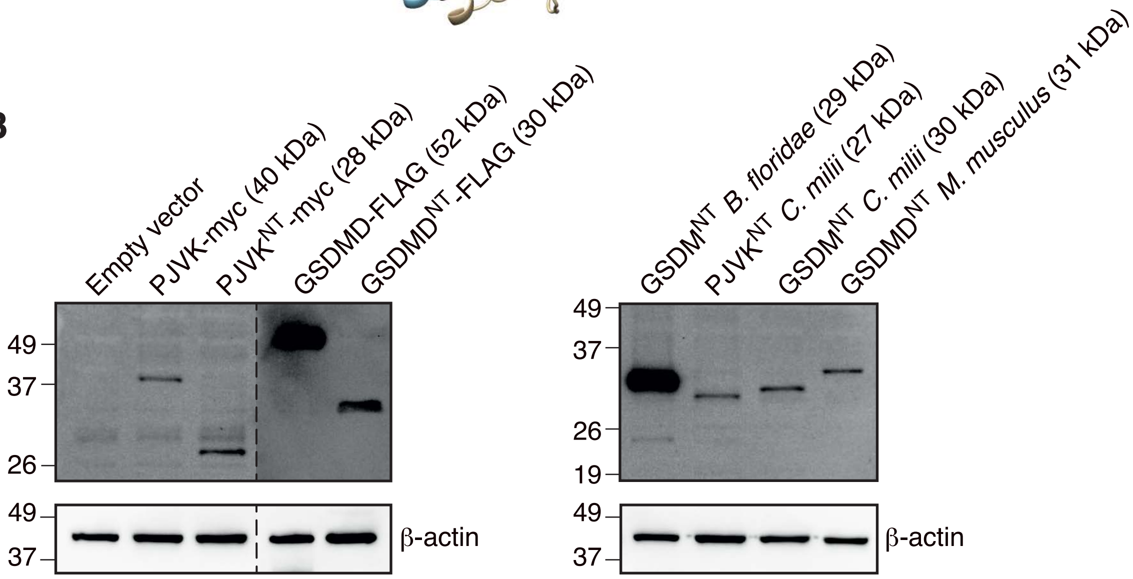

C

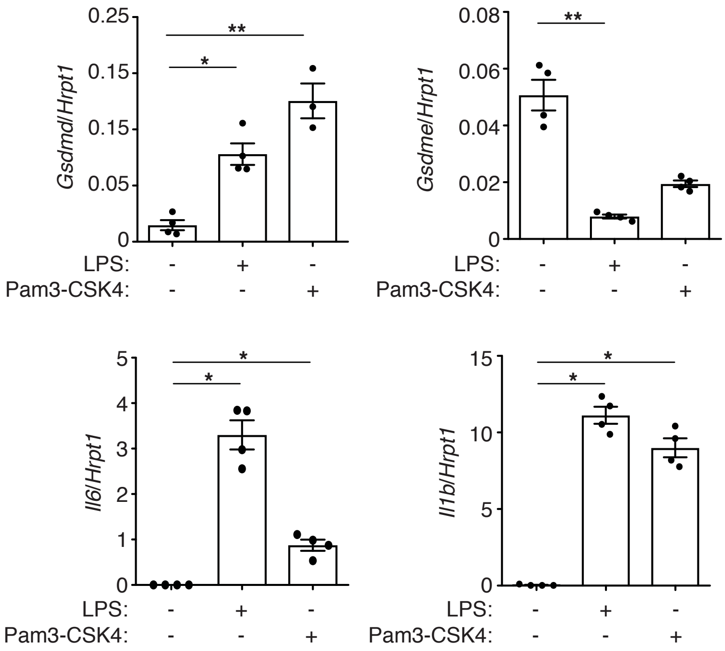

Figure S6. Molecular models of the full-length human PJVK and GSDME proteins. (**A**) Structural alignment of human GSDME (brown) and human PJVK (Cyan) showing 27.1% similarity. Green represents the first α-helix of the N-terminal domain. Both gasdermins have been generated using an intensive modelling mode with Phyre^2 software. (**B**) Immunoblot for anti-myc (to detect myc tagged *H. sapiens* PJVK and PJVK^{NT}, *B. floridae* GSDM^{NT}, *C. milii* PJVK^{NT} and GSDM^{NT}, and *M. musculus* GSDMD^{NT}), anti-FLAG (to detect FLAG tagged *H. sapiens* GSDMD and GSDMD^{NT}) and β-actin in cell lysates from transfected HEK293; numbers on the left correspond to the molecular weight marker expressed in kDa. (**C**) Expression of *Gsdmd*, *Gsdme*, *Il6* and *Il1b* from mouse bone marrow derived macrophages by quantitative PCR after incubation for 4h with LPS or Pam3-CSK4.
