# Supplementary material for: Evolutionary analyses of the gasdermin family suggest conserved roles in infection response despite loss of pore-forming functionality
Source: BMC Biol. 2022 Jan 7;20:9. doi: 10.1186/s12915-021-01220-z (PMC8742441; doi:10.1186/s12915-021-01220-z)
Supplement: Supplementary file 4 — Additional file 4: Fungal regulated cell death (RCD) sequences used in this study. [file 12915_2021_1220_MOESM4_ESM.docx]

**Additional file 4:**

Fungal regulated cell death (RCD) sequences used in this study.

Sequences were obtained from the available deposited data set (<https://doi.org/10.6084/m9.figshare.10298045>) belonging to the Daskalov A *et al*. publication [39]. The authors used as initial query the *rcd-1-1* sequence from *N. crassa* to obtain *rcd-1* homologs.

**Nucleotide *rcd* sequences:**

>Ncra-P4471_g8802

ATGGACAACGAAGAGTGGTTCCCTCTCAAACAAACCCACTACCCACCACCGACCATCCCATCCATGAAAACAGGCCGTCCCACCGGTCCCATCTCTATAGGCCACATCATCCCCGACCTCCGCCACCTCGACAATGTTATCAACTGCAAGGGTTTCGAGCCCTTCCCTCCCAACATGGACGTCTTCACCGCCCACTACGAACAGTGCCACTTCGGGGACCACCTTAACTCCGAGTTCGTCGTGCAAGCCAAGGCCGCAGCGCCCATAAAGAATTTCGTCCCCGGCGTGGACGTCAGTGGTAGCGCTGGGATACATCATACAAATATTACTAGTGATCGTTGGGAGTATGACAGTGTGGTGGAGTACGCGGTGTACCCGACGAGGCAGTATATCGACCGGTTGTTAGAGTCGAAGGAGGTGAGGCAGTATATACAGAAGAGCAAGAAGCTGTTGGGCGGGTGGTGCGTGTATATGGTGACGGGAATCATGGTGGCGAGGGGAGGGGGGAGGAATGTGGTGAGTGAGGAGAAGGGGGCGGGGGTGTTTTCCAATGTTGGCTTCCAGGTACCGGGAATCGGGGAATTTGCGCCAGAAGTGGGATGGGACACGAAGACCAAGACAAAGGTGAATGCTCATCATACAACGGACTTTGTATGTGCGATACGGCTGGTGAAGATTGCAAAGAGTGGGTTGAGATCGAGCTGGACGATGAAGAAAGTAACTAGGGAATTC

>Ndis-PS6-FGSC9959_g5823

ATGGAGAAATGCTGGTTCAACGTCCGCGACACTCACCTCCCACCACCTTCCCTCGACAGCATGCGCTCCGGCCACCCTTCCTCCCTCACCTCCCTGGGCCATCTCATCCCCTCCCTCAACCAATTTGACCAAATCATCAATGCTGATGCCTTGGAGCCCTGCCCACCCACCATGGAAATATACGGTCCCACCGTGATAGAAGAATTCACCTGGGACCACTCGCTCACGCACTCGCTCACGCTAAAAGCCAAAGTCGGCGCCCCCCTTGCCGCGGCCGGCATCCCCGTTCTGAAAGTGAACGTCGGTGCGGGAGGGGCGTTCAGCAAGAGCGTAGGCAACTACTGGGAATTTGATCGGCTGGAGAGGTACATCATGCAGCCGACGAGGAGTTATGTGCAGAAATGTCTTGAGAGGGATGATGTGAAGCAGTGGATTGCCAAAAACAAGTGGATGGGTGGGTGGGAGATGTACATGATTACGGGCATCATTGTGGCCAGGGGAGGGGGCAGGAAGAAAGAGGAGAAGAAGAGGGGGAAAGAGCTGTCCGTGGAGGTTACAATGGAAGTGCCGATCATCATTGAGGCTGGGCCAGAGGTCAAGAAGAATATCACGAGGCAGACGACTTGGGGTGGGGGTCTGACTCATGACTTTGTTTGGGCAGTGAGGCTGGCTAAGATCACCAAACACGGATTGCAGTCGGACTGGAAGATGGAGACGGTTTTTGGAAAGGCATCGTTCCGCGGGCAAAAGGCAATA

>Ncra-JW242_g8226

ATGGATAAATGCTGGTTCACCCTCGACAACGCACACTACCCACCGCCTTCCCTCGACAGCATGCGTTCCGGCCACGCCATCTCGCCCGCCTCCCTAGGCCATCTCATCCCCTCCCTCGCCCATCTCGACCAAATCATCAACGCCAATGCCATCGAGCCCTTCCCATCCACCATGGATATCCACGGCCCCACCATAATAGAAGACTTCAAGTGGGACCACTCCCACACACACTCGCTCTCGCTAGAAGGCAAAGCCTCTATTCCCCTCGGCCCGGCTGGTGTGAACATGAGCGTCGGTTTGGGAGGGGCCTTCAGCAGGAGCGTAGCCAATTACTGGGAGTTCGACCGGCTCGAGAGGTACATTATGCAGCCGACGAGGAGTTATGTGCAGAAATGTATTGAGCGGGACGAGGTGAAGCGGTGGATTGCAAAGAACAAGAGCATGATGATGATGGGCAGGTGGGAGGTGTACATGATTACA

GGCATCATTGTGGCGAGGGGAGGCGGCAGGAAGAAGAAGGAGAAGACGACGGGGAAGGAGTTTTCGGTGGAGGTTACAGTGGAAGTGCCGCTTATCGTTGAGGCTGGACCAGGGGGTAAGAGGAACATGGCGAGGCAGAAGACGTGGGGTACGAGTCAGACGGGTGACTTTGTCTGGGCAGTGAGGTTGGCTAAAATCACTAAGAGCGGGTTGCACTCGGACTGGAAGATGGAGACGGTGTTTGGAAAGACATCATCATTTCGCGGGCAAAAGGCCATTTTC

>Ndis-PS5-FGSC9962_g7383

ATGGAGAAATGCTGGTTCAACGTCCGCGACACTCATCTCCCACCACCATCCCTCGACAGCATGCGCTCCGGCCACCCTTCCTCCCCCACCTCCCTGGGTCATCTCATCCCTTCCCTCAACCAAATCGACAAAATCATCAACGCTGATGCCTTGGAGCCCTTCCCACCCACCATGGAAATATACGGTCCCACAGTGATAGAAGAATTTACCTGGGACCACTCGCTCACCCACTCGCTCACGCTAAAAGCCAAAGTCGGCGCCCCCCTTGCCGCGGCTGGCATCCCCGTTCTGAAAGTGAACGTCGGTGCGGGAGGGGAGTTCAGCAAGAGCGTAGGCAACTACTGGGAATTTGATCGGCTGGAGAGGTACATTATGCAGCCGACGGAGAGTTATGTGCAGAGATGTCTTGAGAGGGATGATGTGAAGCAGTGGATTGCCAAAAACAAGTGGATGGGTGGGTGGGAGATGTACATGATCACG

GGCATCATTGTGGCCAGAGGAGGGGGCAAGAAGAAAGAGGAAAAGAAGAGGGGGAAAGAGCTGTCCGTGGAGGTTACAGTGGAAGTGCCGATCATCATTGAGGCTGGACCAGAGGTTAAGAAGAATATCACGAGGCAGACGACTTTGGGTGGGGGTCTGACGAATGACTTTGTTTGGGCAGTGAGGCTGGCTAAGATCACCAAACACAGATTGCAGTCGGATTGGAAGATGGAGACGGTTTTTGGAAAGGCATCGTTCCGCGGGCAAAAGGCAATA

>Sorma_000182805.2_ASM18280v2_XP_003350360.1

ATGGAAAAATGCTGGTTCCCCCTCCGAGACACTCACTTCCCACCTCCTTCTCTCGAAAGCATGCGCTCCGGCAGTCCTTCCTCCCCCGCCCCTTCCCTAGGCCATCTCATCCCCTCCCTCAACCGCCTCGACCAGATCATCAACGCCGATGGCTTCGAGCCCTTCCCACCGGCCACCATGGAAATCTGGGGCCCGACACTGATAGAAGACTTTTCCTGGGACCAATCCCTCACGCAAAAATCCACAGTAAAACTCAAAGCCAGCGCTCCCCTCGCCGGGGCCGTCATCCCCGGCGCTGAATTGGGCGTTGGCGTGGGAGCGGCGTTTAGCAAGAGCGTGGGCAATTACTGGGAATTTGATCGGTTGGAGAGATACATAGTGCAGCCGACGAGGAGTTATGTGCAGAGGTGCATTGAGAGGGATGATGTGAAGCGGTGGATTGCGAAGAATAAGAAACTGGGTGGGAGGTGGGAGGTGTACATGGTTACGGGCATTATGGTGGCGAGGGGGGGGAGGGAGGAAGAGGAAACGGGAGACGAGCGAGAAGGGGGTGTTTGCGGAGGTTACGGTGTAAGTTCATTTTCTTTGGAAATCCGACGGCAGTCAAAAAGAGCTCGAGAGTTCGAAAGTTCGAGCCGAGTCAAGTTCGATTCTGGTTCCGGTTCCGGGTCCCGGGCTGAGTCCTGCACC

>Ndis-CAWA-W978-3_g6224

ATGGAGAAATGCTGGTTCAACGTCCGCGACACTCATCTCCCACCACCATCCCTCGACAGCATGCGCTCCGGCCACCCTTCCTCCCCCACCTCCCTGGGTCATCTCATCCCTTCCCTCAACCAAATCGACAAAATCATCAACGCTGATGCCTTGGAGCCCTTCCCACCCACCATGGAAATATACGGTCCCACAGTGATAGAAGAATTTACCTGGGACCACTCGCTCACCCACTCGCTCACGCTAAAAGCCAAAGTCGGCGCCCCCCTTGCCGCGGCTGGCATCCCCGTTCTGAAAGTGAACGTCGGTGCGGGAGGGGAGTTCAGCAAGAGCGTAGGCAACTACTGGGAATTTCATCGGCTGGAAAGGTACATTATGCAGCCGACGGAGAGTTATGTGCAGAGATGTCTTGAGAGGGATGATGTGAAGCAGTGGATTGCCAAAAACAAGTGGATGGGTGGGTGGGAGATGTACATGATCACG

GGCATCATTGTGGCCAGAGGAGGGGGCAAGAAGAAAGAGGAAAAGAAGAGGGGGAAAGAGCTGTCCGTGGAGGTTACAGTGGAAGTGCCGATCATCATTGAGGCTGGACCAGAGGTTAAGAAGAATATCACGAGGCAGACGACTTTGGGTGGGGGTCTGACGAATGACTTTGTTTGGGCAGTGAGGCTGGCTAAGATCACCAAACACAGATTGCAGTCGGATTGGAAGATGGAGACGGTTTTTGGAAAGGCATCGTTCCGCGGGCAAAAGGCAATA

>Ncra-P4452_g6422

ATGGATAAATGCTGGTTCACCCTCGACAACGCACACTACCCACCGCCTTCCCTCGACAACATGCGTTCCGGCCACGCCATCTCGCCCGCCTCCCTAGGCCATCTCATCCCCTCCCTCAACCATCTCGACCAAATAATCAACGCCAAAGCCATCGAGCCCTTCCCACCCACCATGGATATCCACGGTCCCACCATAATAGAAGACTTCAAGTGGGACCACTCCCACGAGTACTCGCTCTCGCTAGGAGGCAAAGTCCCCATTCCCCTCGCCCCGGCTGGTGTCCCCTTTGTGGACCTAAACGTCGGTTTGGGAGGGGCCTTCAGCAGGAGCGTAGCCAATTACTGGGAGTTCGACCGGCTCGAGAGGTACATTATGCAGCCGACGAGAAGTTATGTGCAGAAATGTATTGAGCGGGACGAGGTGAAGCGGTGGATTGCAAAGAACAAGAGCATGATGATGATGGGCAGGTGGGAGGTGTAC

ATGATCACCGGCATCATTGTGGCGAGGGGAGGCGGCAGGAAGAAGAAGGAGAAGACGACGGGGAAGGAGTTTTCGGTGGAGGTTACAGTGGAAGTGCCGCTTATCGTTGAGGCTGGACCAGGGGGTAAGAGGAATACGGCGAGGCAGAAGACGTGGGGTACGAGTCAGACGGGTGACTTTGTCTGGGCAGTGAGGTTGGCTAAAATCACTAAGAGCGGGTTGCACTCGGACTGGAAGATGGAGACGGTGTTTGGAAAGACATCATCATTTCGCGGGCAAAAGGCCATT

>Ncra-P4489_g5773

ATGGATAAATGCTGGTTCACCCTCGACAACGCACACTACCCACCGCCTTCCCTCGACAACATGCGTTCCGGCCACGCCATCTCGCCCGCCTCCCTAGGCCATCTCATCCCCTCCCTCAACCATCTCGACCAAATAATCAACGCCAAAGCCATCGAGCCCTTCCCACCCACCATGGATATCCACGGTCCCACCATAATAGAAGACTTCAAGTGGGACCACTCCCACGAGTACTCGCTCTCGCTAGGAGGCAAAGTCCCCATTCCCCTCGCCCCGGCTGGTGTCCCCTTTGTGGACCTAAACGTCGGTTTGGGAGGGGCCTTCAGCAGGAGCGTAGCCAATTACTGGGAGTTCGACCGGCTCGAGAGGTACATTATGCAGCCGACGAGGAGTTATGTGCAGAAATGTATTGAGCGGGACGAGGTGAAGCGGTGGATTGCAAAGAACAAGAGCATGATGATGATGGGCAGGTGGGAGGTGTAC

ATGATTACAGGCATCATTGTGGCGAGGGGAGGCGGCAGGAAGAAGAAGGAGAAGACGACGGGGAAGGAGTTTTCGGTGGAGGTTACAGTGGAAGTACCGCTTATCGTTGAGGCTGGACCAGGGGGTAAGAGGAATACGGCGAGGCAGACGACGTGGGGTACAAGTCAGACGGGTGACTTTGTTTGGGCAGTGAGGTTGGCTAAAATCACTAAGAGCGGGTTGCACTCGGACTGGAAGATGGAGACGGTGTTTGGAAAGACATCATCATTTCGCGGGCAAAAGGCCATTTTC

>Ncra-D30

ATGGACAACGAAGAGTGGTTCCCTCTCAAACAAACCCACTACCCACCACCGACCATCCCATCCATGAAGACAGGCCATCCCACCGGTCCCATCTCTATAGGCCACATCATCCCCGACCTCCGCCACCTCGACAATGTTATCAACTGCAAGGGTTTCGAGCCCTTTCCTCCCAATATGGACGTCTTCACCGCCCACTACGAACAGTGCCACTTCGGGGACCACCTTAACTCCGAGTTCGTCGTGCAAGCCAAGGCCGCAGCGCCCATAAAGAACATTGTCCCCGGCGTGGATGTCACTGGTAGCGCGGGGTTGCATCATACGAATATTACTAGTGATCGTTGGGAGTATGATAGTGTGGTGGAGTACGCGGTGTACCCGACCAGGCAGTATATCGACCGGTTGTTGGAGTCGAAGGAGGTGAAGCAGTATATACAGAAGAGCAAGAAGCTGTTGGGTGGGTGGTGCGTGTATATGGTGACG

GGAATCATGGTGGCGAGGGGAGGGGGGAGGAATGTGGTGAGTGAGGAGAAGGGGGCGGGGGTGTTTGGGAATGTTGGCTTCCAGGTACCGGGAATCGGGGAANTTGCGCCAGAAGTGGGATGGGACACGAAGACCAAGACAAAGGTGAATGCTCATCATACAACGGACTTTGTATGTGCGATACGGCTGGTGAAGATTGCAAAGAGTGGGTTGAGATCGAGCTGGACGATGAAGAAAGTAACTAGGGAATTC

>Ntet-L4-FGSC7585_g5786

ATGCTGGTTCACCCTCAACAACGCACACTTCCCACCGCCTTCCCTCGTTCCGGCCACCCCATCTCCCCCGCCTCCCTAGGCCATCTCATCCCCTCCCTCAACCATCTCGACCAAATCATCAACGCTGATGCTATCGAGCCCTTCCCACCCACCATGGACATCCACGGTCCCACCATAATAGAAGACTTCAAGTGGGACCACTCCCACGAGTACTCGCTCTCGCTAAGAGGCAAAGTCCCCATCCCCCTCGCCGCGGCTGGTGTCCCCTTTGTGGACCTGGGCGTCGGTTCGGGAGGGGCCTTCAGCAAGAGTGTAGCCAATTACTGGGAATTCGACCGGCGGGAGAGGTACATTATGCAGCCGACGAGGAGTTATGTGCAGAAATGTATTGAGCGGGAAGAGGTGAAGCGGTGGATTGCA

AAGAACAAGAGCATGATGATGATGGGCAGGTGGGAGGTGTACATGATTCACAGGCATCATTGTGGCGAGGGGAGGGGGCAGGAAGACGAAGGAGAAGAAGACGGGGAAGGAGTTTTCGGTGGAGGTTACAGTGTAAGTTCCATCCCTTTCGCTGAACCGCAGCCAGCACCCGGAAAGTCCCGAGAGTTCGTCGCGAGTCCTGTCGTCGGGGAAGTGCCGCTTATTGTTGAGGCTGGGCCGGGGGTCAAGAGGAACATGACGAGGCAGACGACGTGGGGTACGAGTCAGACGCATGACTTTGTTTGGGCAGTGAGGTTGGCTAAAATCACTAAGAGCGGGTTGCACTCGGACTGGAAGATGGAGACGGTGTTTGGAAAGACATCGTTTCGCGGGCAAAAGGCCATT

>Ntet-L8-FGSC9043_g8716

ATGGATAAATGCTGGTTCACCCTCAACAACGCACACTTCCCACCGCCTTCCCTCGACAGCATGCGTTCCGGCCACCCCATCTCCCCCGCCTCCCTAGGCCATCTCATCCCCTCCCTCAACCATCTCGACCAAATCATCAACGCTGATGCCATCGAGCCCTTCCCACCCACCATGGACATCCACGGTCCCACCATAATAGAAGACTTCAAGTGGGACCACTCCCACGAGTACTCGCTCTCGCTAAGAGGCAAAGTCCCCATCCCCCTCGCCGCGGCTGGTGTCCCCTTTGTGGACCTGGGCGTCGGTTTGGGAGGGGCCTTCAGCAAGAGCGTAGCCAATTACTGGGAATTCGACCGGCTGGAGAGGTACATTATGCAGCCGACGAGGAGTTATGTGCAGAAATGTATTGAGCGGGAAGAGGTGAAGCGGTGGATTGCAAAGAACAAGAGCATGATGATGATGGGCAGGTGGGAGGTGTACATGATTCACAGGCATCATTGTGGCGAGGGGAGGGGGCAGGAAGACGAAGGAGAAGAAGACGGGGAAGGAGTTTTCGGTGGAGGTTACAGTGTAAGTTCCATCCCTTTCGCTGAACCGCAGCCAGCACCCGGAAAGTCCCGAGAGTTCGTCGCGAGTCCTGTCGTCGGGGAAGTGCCGCTTATCGTTGAGGCTGGGCCGGGGGTCAAGAGGAACATGACGAGGCAGACGACGTGGGGTACGAGTCAGACGGATGACTTTGTTTGGGCAGTGAGGTTGGCTAAAATCACTAAGAGCGGGTTGCACTCGGACTGGAAGATGGAGACGGTGTTTGGAAAGACATCGTTTCGCGGGCAAAAGGCCATT

>Ntet-L8-FGSC9028_g3224

ATGGATAAATGCTGGTTCACCCTCAACAACGCACACTTCCCACCGCCTTCCCTCGACAGCATGCGTTCCGGCCACCCCATCTCCCCCGCCTCCCTAGGCCATCTCATCCCCTCCCTCAACCATCTCGACCAAATCATCAACGCTGATGCCATCGAGCCCTTCCCACCCACCATGGACATCCACGGTCCCACCATAATAGAAGACTTCAAGTGGGACCACTCCCACGAGTACTCGCTCTCGCTAAGAGGCAAAGTCCCCATCCCCCTCGCCGCGGCTGGTGTCCCCTTTGTGGACCTGGGCGTCGGTTTGGGAGGGGCCTTCAGCAAGAGCGTAGCCAATTACTGGGAATTCGACCGGCTGGAGAGGTACATTATGCAGCCGACGAGGAGTTATGTGCAGAAATGTATTGAGCGGGAAGAGGTGAAGCGGTGGATTGCAAAGAACAAGAGCATGATGATGATGGGCAGGTGGGAGGTGTACATGATTCACAGGCATCATTGTGGCGAGGGGAGGGGGCAGGAAGACGAAGGAGAAGAAGACGGGGAAGGAGTTTTCGGTGGAGGTTACAGTGTAAGTTCCATCCCTTTCGCTGAACCGCAGCCAGCACCCGGAAAGTCCCGAGAGTTCGTCGCGAGTCCTGTCGTCGGGGAAGTGCCGCTTATCGTTGAGGCTGGGCCGGGGGTCAAGAGGAACATGACGAGGCAGACGACGTGGGGTACGAGTCAGACGGATGACTTTGTTTGGGCAGTGAGGTTGGCTAAAATCACTAAGAGCGGGTTGCACTCGGACTGGAAGATGGAGACGGTGTTTGGAAAGACATCGTTTCGCGGGCAAAAGGCCATT

>Nter-FGSC1889_g2628

ATGGACAACGAAGCCTGGCTCCCTCTTAAACAATCCCACTACCCACCACCGTCCATTCCATCTATGAAGACAGGCTGTCCAACCGGTCCCATCTCTCCAGGTCACATCATCCCCGACCTCCGACACCTCGACAACGTCATCAACTGCAATGGCTTCGAGCCCTTTCCCTCCGGGATGGACATCATGACCGCCACCCTCGAACAGAGCACCTTCAAGAACGGCGTCAACACCGAGATAGTCCTCCAAGCCAAGGCCGAAGCACCAATAGCGGGTATGATTCCCGGCGTGGATGCTAGTGCTGGTGCTGGGACCAATTACACGAATATTATCAGTGACAGTTGGGAGTATGAACGTTTGGAAGAGTACGTGGTGCAGCCTACGAGGCAGTATGTCCAGCGGTGTTTGGAGTCAGAGGAGGTGGCGGGGTATATACAGAGGAGTAAGAAGTTGGGAGGTTGTGAGGTGCCGGGAATCGGGGAA

TTCGCGCCAGAAGTTGGATGGGATAAGAAGACCAAGTCGACAGTGAACGGTCATCATACGGCGGACTTTGTGTGCGCCATTCGGCTGGTGAAGATTACGAAGAGTGGGTTGAGATCGAGT

TGGACGATGAAGACGGTGACTAGG

>Ndis-PS9-FGSC9953_g12136

ATGGACAACGAAGCCTGGTTCCCTCTCAAACAATCCCACTACCCTCCACCGTCCATTCCATCCATGAAAACAGGCCATCCAACCGGTCCCATCTCTCCAGGACACATCATCCCCGACCTCCGACACCTCGACAACGTCATCAACTGCAATGGCTTCGAGCCCTTTCCAGCCAGCATGGACATCATGAGCGCCACTTTTGAACAGTGCACCTTCAAGGACGGCCTTAACTCAGAGTTCGTCCTGCAAGCCAAGGCCGAAGCACCCATAACGAGTATTGCTCCCGGCGTGGATGTCACTGGTAGCGCTGGGATGAACTATACCAATATAGTCAGTGATAGCTGGGAGTATGAAGGTCTGGAGGAGCACGTGGTGCAGCCGACGAGGCAGTATATCGAGCGGTGTTTGGAGTCCGAGGAGGTGGCGGAGTATACACAGAAGAGTAAGAAGCTGTTGGGAGGCTGGTGCGTGTATATGATTACG

GGAATCATGGTGGCTAAGGGAGGGGGGAAGAATGTGGTGAGTGAGGAGAAGGGGGTGGGGGTGTTTGGGAATATTGGCTTCGAGGTGCCGGGAATCGCGGAGTCTGCGCCAGAATTCGGATGGGATAAGAGGGCCAAGTCGACGGTGAATGGTCATCATACGACGGACTTTGTATGTGCAATACGGCTGGTGAAGATCGCGAAGAGCGGATTGAGATCGAGTTGGACGATGAAGACAGTGACTAGG

>Ncra-JW75_g1889

ATGGATAAATGCTGGTTCACCCTCGACAACGCACACTACCCACCGCCTTCCCTCGACAGCATGCGTTCCGGCCACGCCATCTCGCCCGCCTCCCTAGGCCATCTCATCCCCTCCCTCGCCCATCTCGACCAAATCATCAACGCCAATGCCATCGAGCCCTTCCCATCCACCATGGATATCCACGGCCCCACCATAATAGAAGACTTCAAGTGGGACCACTCCCACACACACTCGCTCTCGCTAGAAGGCAAAGCCTCTATTCCCCTCGGCCCGGCTGGTGTGAACATGAGCGTCGGTTTGGGAGGGGCCTTCAGCAGGAGCGTAGCCAATTACTGGGAGTTCGACCGGCTCGAGAGGTACATTATGCAGCCGACGAGGAGTTATGTGCAGAAATGTATTGAGCGGGACGAGGTGAAGCGGTGGATTGCAAAGAACAAGAGCATGATGATGATGGGCAGGTGGGAGGTGTACATGATCACC

GGCATCATTGTGGCGAGGGGAGGCGGCAGGAAGAAAGAGGAGAAGAAGACGGGGAAGGAGTTTTCGGTGGAGGTCACTGTGGAAGTGCCGCTTATCGTTGAGGCTGGACCAGGGGGTAAGAGGAATACGACGAGGCAGAAAACTTGGGGTACAAGTCAGACGGGTGACTTTGTCTGGGCAGTGAGGTTGGCTAAAATCACTAAGAGCGGGTTGCACTCGGACTGGAAGATGGAGACGGTGTTTGGAAAGACATCATCATTTCGCGGGCAAAAGGCTATT

>Ndis-PS10-FGSC9958_g6857

ATGGACAACGAAGCCTGGCTCCCTCTTAAACAATCCCACTACCCTCCACCGTCCATTCCGTCCATGAAAACAGGCCATCCAACCGGTCCCATCTCCCCAGGCCACATCATCCCTGACCTCCGACACCTCGACAACGTCATCAACTGCAATGGCTTCGAACCCTTTCCACCCAGCATGGCCATCATGACGGCTACGTTCGAACAGAGCACCTTCAAGGACGGCGTTAACACAGAGATAGTCCTCCAAGCCAAGGCCGAAGCACCAATAACGAGTATGATCCCCGGCGTGGATGTCACTGCTGGCGCTGGGACGAATTACACGAATATTGTCAGTGACAGTTGGGAGTATGAAGGTCTGGAAGAGTACGTGGTGCAGCCTACGAGGCAGTATATCCAGCGGTGTTTGGAGTCAGAGGAGGTGGCGGCGTATATACAGAAGAGTAAGAAGCTGGGAGGCTGGTGCGTGTATATGATTACGGGA

ATCATGGTGGCAAGGGGAGGGGGGAAGAATGTGGTGAGTGAGGAGAAGGGGGTGGGGTTGTTTGGGAATGTTAACTTCGAGGTGCCGGTAATCGCGGAATTCGCGCCAGAAGTCGGATGGGATAAGAAGACCAAGTCGACGGTGAACGGTCATCATACGACGGACTTTGTATGTGCGATACGGCTGGTGAAGATCGCGAAGAGTGGGTTGAGATCGAGTTGGACGATGAAGACAGTGAC

**RCD protein sequences:**

>Ncra-P4471_g8802

MDNEEWFPLKQTHYPPPTIPSMKTGRPTGPISIGHIIPDLRHLDNVINCKGFEPFPPNMDVFTAHYEQCHFGDHLNSEFVVQAKAAAPIKNFVPGVDVSGSAGIHHTNITSDRWEYDSVVEYAVYPTRQYIDRLLESKEVRQYIQKSKKLLGGWCVYMVTGIMVARGGGRNVVSEEKGAGVFSNVGFQVPGIGEFAPEVGWDTKTKTKVNAHHTTDFVCAIRLVKIAKSGLRSSWTMKKVTREF

>Ndis-PS6-FGSC9959_g5823

MEKCWFNVRDTHLPPPSLDSMRSGHPSSLTSLGHLIPSLNQFDQIINADALEPCPPTMEIYGPTVIEEFTWDHSLTHSLTLKAKVGAPLAAAGIPVLKVNVGAGGAFSKSVGNYWEFDRLERYIMQPTRSYVQKCLERDDVKQWIAKNKWMGGWEMYMITGIIVARGGGRKKEEKKRGKELSVEVTMEVPIIIEAGPEVKKNITRQTTWGGGLTHDFVWAVRLAKITKHGLQSDWKMETVFGKASFRGQKAI

>Ncra-JW242_g8226

MDKCWFTLDNAHYPPPSLDSMRSGHAISPASLGHLIPSLAHLDQIINANAIEPFPSTMDIHGPTIIEDFKWDHSHTHSLSLEGKASIPLGPAGVNMSVGLGGAFSRSVANYWEFDRLERYIMQPTRSYVQKCIERDEVKRWIAKNKSMMMMGRWEVYMITGIIVARGGGRKKKEKTTGKEFSVEVTVEVPLIVEAGPGGKRNMARQKTWGTSQTGDFVWAVRLAKITKSGLHSDWKMETVFGKTSSFRGQKAIF

>Ndis-PS5-FGSC9962_g7383

MEKCWFNVRDTHLPPPSLDSMRSGHPSSPTSLGHLIPSLNQIDKIINADALEPFPPTMEIYGPTVIEEFTWDHSLTHSLTLKAKVGAPLAAAGIPVLKVNVGAGGEFSKSVGNYWEFDRLERYIMQPTESYVQRCLERDDVKQWIAKNKWMGGWEMYMITGIIVARGGGKKKEEKKRGKELSVEVTVEVPIIIEAGPEVKKNITRQTTLGGGLTNDFVWAVRLAKITKHRLQSDWKMETVFGKASFRGQKAI

>Sorma_000182805.2_ASM18280v2_XP_003350360.1

MEKCWFPLRDTHFPPPSLESMRSGSPSSPAPSLGHLIPSLNRLDQIINADGFEPFPPATMEIWGPTLIEDFSWDQSLTQKSTVKLKASAPLAGAVIPGAELGVGVGAAFSKSVGNYWEFDRLERYIVQPTRSYVQRCIERDDVKRWIAKNKKLGGRWEVYMVTGIMVARGGREEEETGDEREGGVCGGYGVSSFSLEIRRQSKRAREFESSSRVKFDSGSGSGSRAESCT

>Ndis-CAWA-W978-3_g6224

MEKCWFNVRDTHLPPPSLDSMRSGHPSSPTSLGHLIPSLNQIDKIINADALEPFPPTMEIYGPTVIEEFTWDHSLTHSLTLKAKVGAPLAAAGIPVLKVNVGAGGEFSKSVGNYWEFHRLERYIMQPTESYVQRCLERDDVKQWIAKNKWMGGWEMYMITGIIVARGGGKKKEEKKRGKELSVEVTVEVPIIIEAGPEVKKNITRQTTLGGGLTNDFVWAVRLAKITKHRLQSDWKMETVFGKASFRGQKAI

>Ncra-P4452_g6422

MDKCWFTLDNAHYPPPSLDNMRSGHAISPASLGHLIPSLNHLDQIINAKAIEPFPPTMDIHGPTIIEDFKWDHSHEYSLSLGGKVPIPLAPAGVPFVDLNVGLGGAFSRSVANYWEFDRLERYIMQPTRSYVQKCIERDEVKRWIAKNKSMMMMGRWEVYMITGIIVARGGGRKKKEKTTGKEFSVEVTVEVPLIVEAGPGGKRNTARQKTWGTSQTGDFVWAVRLAKITKSGLHSDWKMETVFGKTSSFRGQKAI

>Ncra-P4489_g5773

MDKCWFTLDNAHYPPPSLDNMRSGHAISPASLGHLIPSLNHLDQIINAKAIEPFPPTMDIHGPTIIEDFKWDHSHEYSLSLGGKVPIPLAPAGVPFVDLNVGLGGAFSRSVANYWEFDRLERYIMQPTRSYVQKCIERDEVKRWIAKNKSMMMMGRWEVYMITGIIVARGGGRKKKEKTTGKEFSVEVTVEVPLIVEAGPGGKRNTARQTTWGTSQTGDFVWAVRLAKITKSGLHSDWKMETVFGKTSSFRGQKAIF

>Ncra-D30

MDNEEWFPLKQTHYPPPTIPSMKTGHPTGPISIGHIIPDLRHLDNVINCKGFEPFPPNMDVFTAHYEQCHFGDHLNSEFVVQAKAAAPIKNIVPGVDVTGSAGLHHTNITSDRWEYDSVVEYAVYPTRQYIDRLLESKEVKQYIQKSKKLLGGWCVYMVTGIMVARGGGRNVVSEEKGAGVFGNVGFQVPGIGEXAPEVGWDTKTKTKVNAHHTTDFVCAIRLVKIAKSGLRSSWTMKKVTREF

>Ntet-L4-FGSC7585_g5786

MLVHPQQRTLPTAFPRSGHPISPASLGHLIPSLNHLDQIINADAIEPFPPTMDIHGPTIIEDFKWDHSHEYSLSLRGKVPIPLAAAGVPFVDLGVGSGGAFSKSVANYWEFDRRERYIMQPTRSYVQKCIEREEVKRWIAKNKSMMMMGRWEVYMIHRHHCGEGRGQEDEGEEDGEGVFGGGYSVSSIPFAEPQPAPGKSREFVASPVVGEVPLIVEAGPGVKRNMTRQTTWGTSQTHDFVWAVRLAKITKSGLHSDWKMETVFGKTSFRGQKAI

>Ntet-L8-FGSC9043_g8716

MLVHPQQRTLPTAFPRSGHPISPASLGHLIPSLNHLDQIINADAIEPFPPTMDIHGPTIIEDFKWDHSHEYSLSLRGKVPIPLAAAGVPFVDLGVGSGGAFSKSVANYWEFDRRERYIMQPTRSYVQKCIEREEVKRWIAKNKSMMMMGRWEVYMIHRHHCGEGRGQEDEGEEDGEGVFGGGYSVSSIPFAEPQPAPGKSREFVASPVVGEVPLIVEAGPGVKRNMTRQTTWGTSQTHDFVWAVRLAKITKSGLHSDWKMETVFGKTSFRGQKAI

>Ntet-L8-FGSC9028_g3224

MDKCWFTLNNAHFPPPSLDSMRSGHPISPASLGHLIPSLNHLDQIINADAIEPFPPTMDIHGPTIIEDFKWDHSHEYSLSLRGKVPIPLAAAGVPFVDLGVGLGGAFSKSVANYWEFDRLERYIMQPTRSYVQKCIEREEVKRWIAKNKSMMMMGRWEVYMIHRHHCGEGRGQEDEGEEDGEGVFGGGYSVSSIPFAEPQPAPGKSREFVASPVVGEVPLIVEAGPGVKRNMTRQTTWGTSQTDDFVWAVRLAKITKSGLHSDWKMETVFGKTSFRGQKAI

>Nter-FGSC1889_g2628

MDNEAWLPLKQSHYPPPSIPSMKTGCPTGPISPGHIIPDLRHLDNVINCNGFEPFPSGMDIMTATLEQSTFKNGVNTEIVLQAKAEAPIAGMIPGVDASAGAGTNYTNIISDSWEYERLEEYVVQPTRQYVQRCLESEEVAGYIQRSKKLGGCEVPGIGEFAPEVGWDKKTKSTVNGHHTADFVCAIRLVKITKSGLRSSWTMKTVTR

>Ndis-PS9-FGSC9953_g12136

MDNEAWFPLKQSHYPPPSIPSMKTGHPTGPISPGHIIPDLRHLDNVINCNGFEPFPASMDIMSATFEQCTFKDGLNSEFVLQAKAEAPITSIAPGVDVTGSAGMNYTNIVSDSWEYEGLEEHVVQPTRQYIERCLESEEVAEYTQKSKKLLGGWCVYMITGIMVAKGGGKNVVSEEKGVGVFGNIGFEVPGIAESAPEFGWDKRAKSTVNGHHTTDFVCAIRLVKIAKSGLRSSWTMKTVTR

>Ncra-JW75_g1889

MDKCWFTLDNAHYPPPSLDSMRSGHAISPASLGHLIPSLAHLDQIINANAIEPFPSTMDIHGPTIIEDFKWDHSHTHSLSLEGKASIPLGPAGVNMSVGLGGAFSRSVANYWEFDRLERYIMQPTRSYVQKCIERDEVKRWIAKNKSMMMMGRWEVYMITGIIVARGGGRKKEEKKTGKEFSVEVTVEVPLIVEAGPGGKRNTTRQKTWGTSQTGDFVWAVRLAKITKSGLHSDWKMETVFGKTSSFRGQKAI

>Ndis-PS10-FGSC9958_g6857

MDNEAWLPLKQSHYPPPSIPSMKTGHPTGPISPGHIIPDLRHLDNVINCNGFEPFPPSMAIMTATFEQSTFKDGVNTEIVLQAKAEAPITSMIPGVDVTAGAGTNYTNIVSDSWEYEGLEEYVVQPTRQYIQRCLESEEVAAYIQKSKKLGGWCVYMITGIMVARGGGKNVVSEEKGVGLFGNVNFEVPVIAEFAPEVGWDKKTKSTVNGHHTTDFVCAIRLVKIAKSGLRSSWTMKTV
