# Supplementary material for: Evolutionary analyses of the gasdermin family suggest conserved roles in infection response despite loss of pore-forming functionality
Source: BMC Biol. 2022 Jan 7;20:9. doi: 10.1186/s12915-021-01220-z (PMC8742441; doi:10.1186/s12915-021-01220-z)
Supplement: Supplementary file 5 — Additional file 5: Primer sequences data. [file 12915_2021_1220_MOESM5_ESM.pdf]

Additional file 5. Sequences of the oligonucleotides used in this study.

Gen	Name	Sequence (5' to 3')
Gsdma	FM1_Gsdma	GAAGGAAGTTCATATTGATCC
	RM1_Gsdma	ACTGGACTTAACTTCTCCAC
Gsdme	FM1_Dfna5	CCTGTTTGATGAAGAACTCC
	RM1_Dfna5	CTCTCCTTGTATCCTGTATCC
Gsdmd	FM1_Gsdmd	GCTCTAAATGGGATATCCTTC
	RM1_Gsdmd	AATTCCTCCTCATCAATCCC
Gsdmc	FM1_Gsdmc	CCATCTAGATTTCATGTGCC
	RM1_Gsdmc	ATACAGAACATCCCTGTCAC
Pjvk	FM1_Dfnb59	CTTCGAAAGAAACAGGAGAG
	RM1_Dfnb59	GTCGTAGAAGTCAGAAAAGAG
Il1b	FM2_Il1b	GTGATATTCTCCATGAGCTTTG
	RM2_Il1b	TCTTCTTTGGGTATTGCTTG
Il6	FM1_Il6	AAGAAATGATGGATGCTACC
	RM1_Il6	GAGTTTCTGTATCTCTCTGAAG
Hprt1	FM1_Hprt1	AGGGATTTGAATCACACGTTTG
	RM1_Hprt1	TTTACTGGCAACATCAACAG
