# Supplementary figures and images for: Evolutionary analyses of the gasdermin family suggest conserved roles in infection response despite loss of pore-forming functionality
Source: BMC Biol. 2022 Jan 7;20:9. doi: 10.1186/s12915-021-01220-z (PMC8742441; doi:10.1186/s12915-021-01220-z)

Additional File 8. Uncropped membranes Supplementary Figure S6

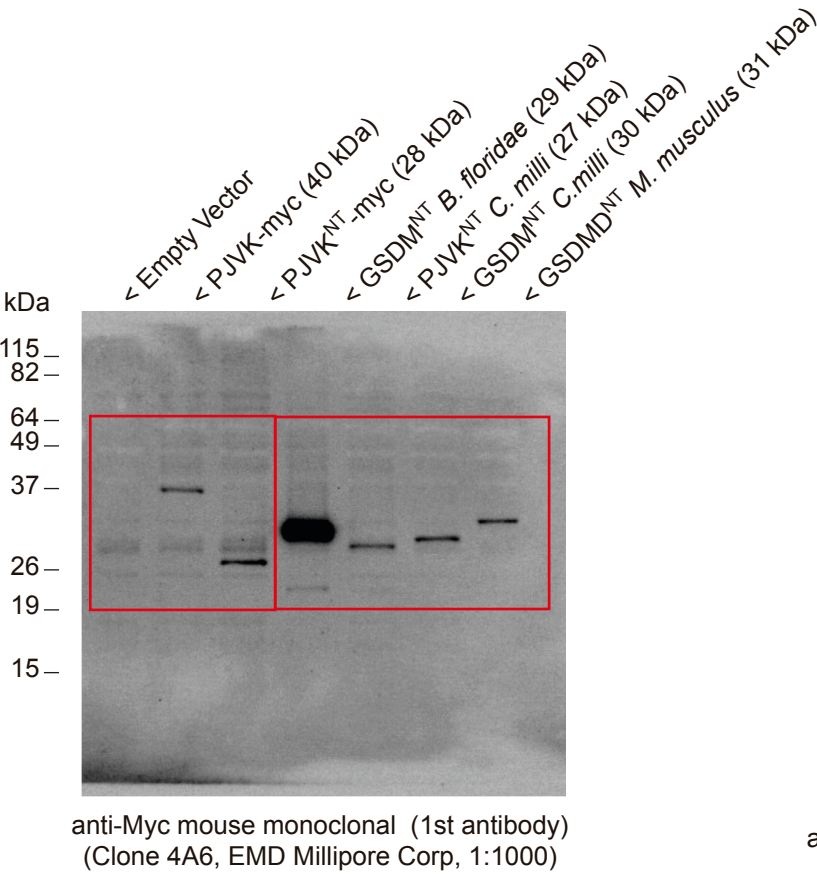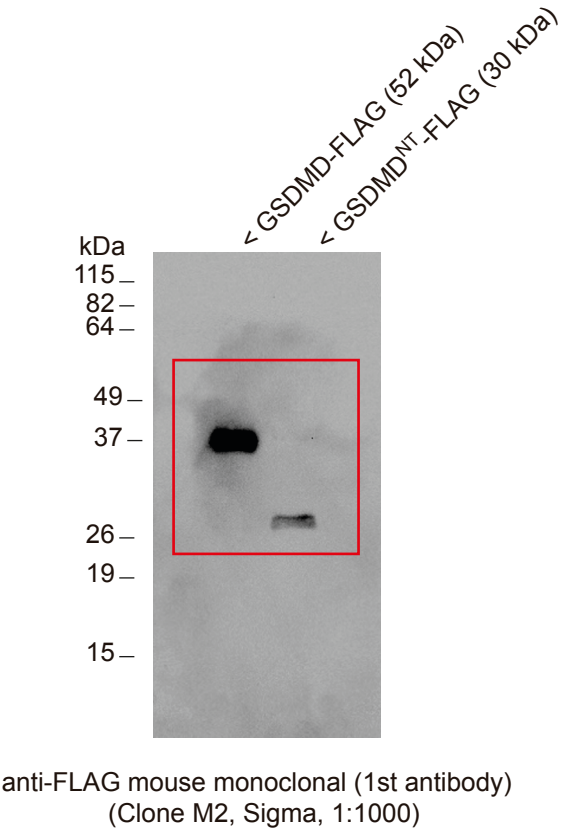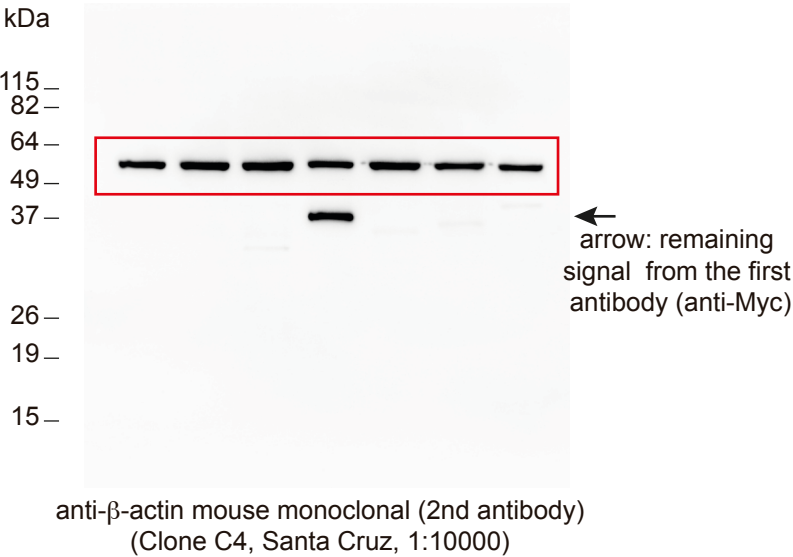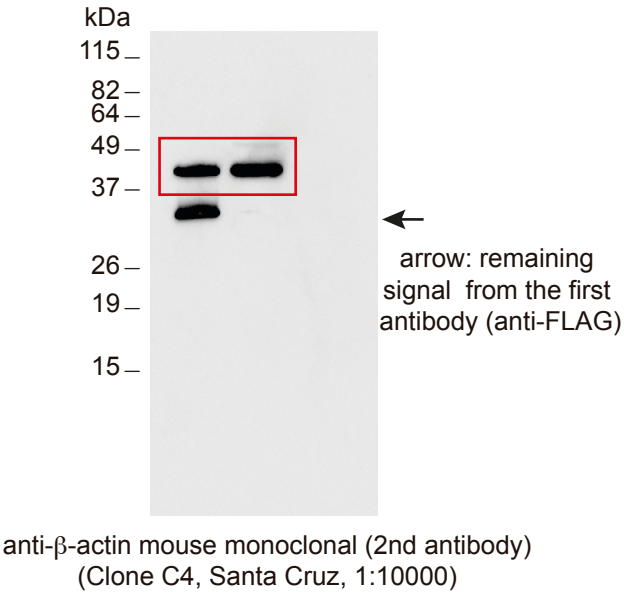

Supplement: Supplementary file 8 — Additional file 8: Uncropped Western blots. [file 12915_2021_1220_MOESM8_ESM.pdf]
